# Oscillations in working memory and neural binding: A mechanism for multiple memories and their interactions

**DOI:** 10.1371/journal.pcbi.1006517

**Published:** 2018-11-12

**Authors:** Jason E. Pina, Mark Bodner, Bard Ermentrout

**Affiliations:** 1 Department of Mathematics, University of Pittsburgh, Pittsburgh, Pennsylvania, United States of America; 2 MIND Research Institute, Irvine, California, United States of America; Plymouth University, UNITED KINGDOM

## Abstract

Neural oscillations have been recorded and implicated in many different basic brain and cognitive processes. For example, oscillatory neural activity has been suggested to play a role in binding and in the maintenance of information in working memory. With respect to the latter, the majority of work has focused primarily on oscillations in terms of providing a “code” in working memory. However, oscillations may additionally play a fundamental role by enabling or facilitating essential properties and behaviors that neuronal networks must exhibit in order to produce functional working memory and the processes it supports, such as combining items in memory into bound objects or separating bound objects into distinct items. In the present work, we present a biologically plausible working memory model and demonstrate that specific types of stable oscillatory dynamics that arise may play critical roles in providing mechanisms for working memory and the cognitive functions that it supports. Specifically, these roles include (1) enabling a range of different types of binding, (2) both enabling and limiting capacities of bound and distinct items held active in working memory, and (3) facilitating transitions between active working memory states as required in cognitive function. Several key results arise within the examinations, such as the occurrence of different network capacities for working memory and binding, differences in processing times for transitions in working memory states, and the emergence of a combinatorially rich and complex range of oscillatory states that are sufficient to map onto a wide range of cognitive operations supported by working memory, such as variable binding, reasoning, and language. In particular, we show that these oscillatory states and their transitions can provide a specific instantiation of current established connectionist models in representing these functions. Finally, we further characterize the dependence of the relevant oscillatory solutions on certain critical parameters, including mutual inhibition and synaptic timescales.

## Author summary

Working memory is a form of limited-capacity short term memory that is relevant to cognition. Various studies have shown that ensembles of neurons oscillate during working memory retention, and cross-frequency coupling (between, e.g., theta and gamma frequencies) has been conjectured as underlying the observed limited capacity. Binding occurs when different objects or concepts are associated with each other and can persist as working memory representations; neuronal synchrony has been hypothesized as the neural correlate. We propose a novel computational model of a network of oscillatory neuronal populations that captures salient attributes of working memory and binding by allowing for both stable synchronous and asynchronous activity. We find biologically plausible sets of parameters that allow for 3 populations to oscillate asynchronously, consistent with working memory capacity, which has been experimentally found to be limited to perhaps 3–5 items. The oscillatory dynamics we describe may provide a mechanism that can facilitate aspects of working memory, such as maintaining multiple items active at once, creating rich neural representations of memories via binding, and rapidly transitioning activation patterns based on selective inputs. Using numerical continuation methods, we partition parameter space into regions that allow for these relevant oscillatory behaviors and gain further insight by exploring the limit of weak coupling between populations.

## Introduction

The neuronal substrate of working memory is thought to be persistent elevated firing rates of neurons that have been found in numerous physiological and imaging studies across widely-varying scales, from single neurons up to neuronal populations and networks [1–6]. Moreover, for working memory to function properly these populations must be able to be activated rapidly by external or internal input corresponding to aspects of memoranda, that activation must be selective and stable, and the elevated firing rates must be able to return to background levels when the information is no longer needed. Cognitive and behavioral considerations further imply that mechanisms must exist for rapidly transitioning between sequences of active memories, and that multiple (and possibly overlapping) selective populations and networks can be simultaneously active. These factors apply both to the case of neural binding, in which the activity of disparate neuronal populations or networks must be combined and maintained (e.g., corresponding to different aspects of a particular item in memory or working memory task), and for the case in which multiple different items are simultaneously maintained in working memory. In this latter case, there are well established approximate upper limits to the number of separate objects that can be simultaneously maintained. Indeed, one of the fundamental properties of working memory is that it has a limited capacity, possibly limited to three to five objects [7–11].

Binding refers to how items encoded in distinct brain circuits or neural populations can be combined for perception, decisions, and action, and can be partitioned to encompass multiple situations [12]. These different binding types include feature binding, which involves the association of different characteristics to an object and how we make and then unravel these associations, and variable binding, which arises, for example, in language and other symbolic thought (e.g., the binding of a variable name and its value). In every instance, some form of synchronization of neural activity has been proposed as the underlying mechanism (e.g., [12–18]). Binding is a key aspect in working memory, as most objects we encounter, physical or symbolic, are multi-featured. There is also a limited capacity to the number of features that may be represented for objects; however, it is unclear at present exactly how this feature capacity relates to the aforementioned working memory capacity [9, 19, 20].

In many studies, oscillatory dynamics have been identified with cognitive function in general and, increasingly, with working memory in particular (e.g., [21–23]). While the presence of different patterns of oscillations is well documented, the specific roles they play are not well understood. Recent work has suggested, however, that oscillations in various frequency bands and coupled or nested oscillations could play a fundamental role in the functioning of different aspects of working memory [6, 24–27]. Oscillatory dynamics may also play a critical generic role in facilitating the range of dynamics and optimized conditions required for working memory function as described above.

In the present work, the networks are capable of producing a range of oscillatory frequencies implicated in working memory and consistent with these other studies. However, we did not focus on particular frequency bands in terms of their implications for a working memory code, but rather on the role they can play in facilitating the essential functions of working memory. In particular, we examine the role oscillatory states can serve as an underlying mechanism to allow for multiple stable, active items in memory, to establish binding via synchronous relationships, to transition between different active working memory states, and to rapidly activate and terminate activity in those networks as required by the needs of cognition and thought. We show how oscillatory dynamics may facilitate these potentially competing requirements, and identify and discuss critical network parameters involved in achieving these dynamics. Indeed, although all of the network’s parameters play a part in the allowed dynamics, we observed that different parameters played roles of varying importance to each of the states. We thus found it fruitful to first explore some of the features and constraints of the asynchronous and the synchronous states individually, and then to see how these attracting states naturally combine to form more complex activity involving both synchronous and asynchronous oscillations that may represent multiple rich, multi-feature memories. We found that the attracting synchronous and asynchronous states allowed for the representation of bound and distinct items in memory, for multiple items to be bound or to be maintained as distinct, representing different network capacities, and for rapid transitions between the different states as is required during cognition.

## Methods

**Fig 1:**
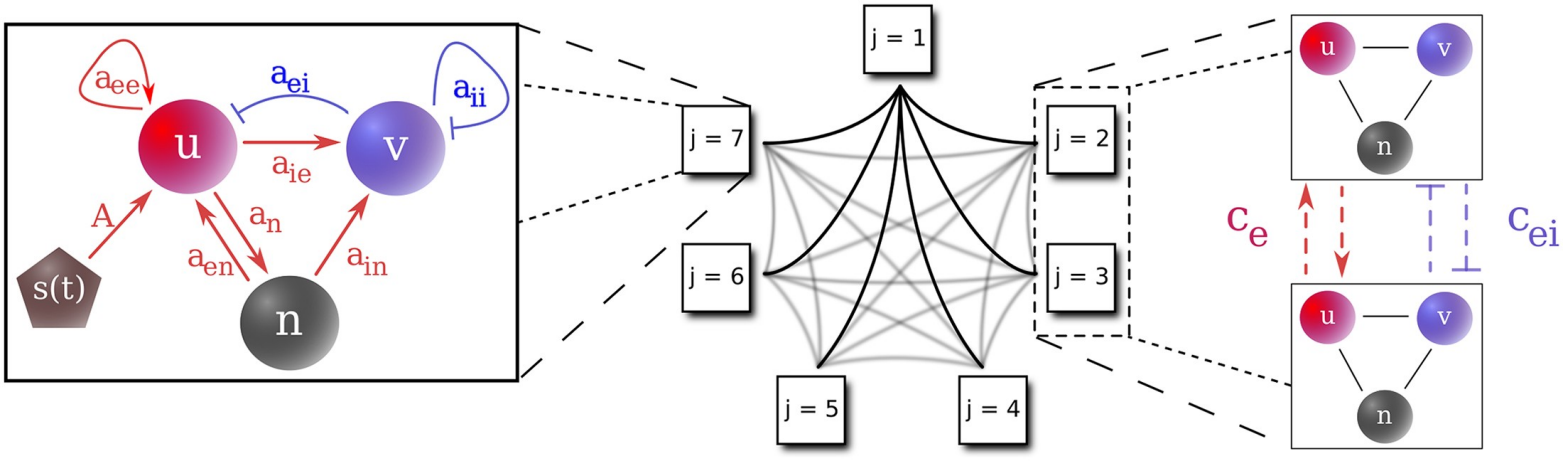
Model connectivity. Left: One population as described in Eq (1). *u* is the fast excitatory AMPA synaptic activity, *v* the inhibitory GABA activity, and *n* the slow excitatory NMDA activity. Feedforward excitation to the AMPA synapses (*u*) triggers activity in the system. Middle: An example network with *N* = 7 populations. The elements are coupled in an all-to-all fashion as shown on the right. Right: Connectivity between two populations, each with three components as shown in the left panel. The populations are connected with excitatory (*c*_*e*_) and inhibitory (*c*_*ei*_) coupling as described in Eq (2).

Oscillations during memory task delays have been seen in population-level activity, including in local field potential recordings and human EEG traces [4, 5]. This motivates us to model working memory oscillatory dynamics at the ensemble level, using Wilson-Cowan type equations for the firing rates (or synaptic activities) of each local circuit that will comprise the model. Here, we provide the outline of the model. In S1 Text we develop the model in greater detail, beginning with a quadratic integrate-and-fire network. Since previous experimental and computational studies suggest NMDA receptors may be crucial to the persistent elevated firing rates associated with proper working memory function in experimental and computational studies, we model the effect of NMDA receptors as a separate component [2, 28–31]:

$$\begin{array}{cc} u_{j}^{\prime } & =-u_{j}+f\left(a_{ee}\cdot {\tilde{u}}_{j}-a_{ei}\cdot {\tilde{v}}_{j}+a_{en}\cdot {\tilde{n}}_{j}-\theta_{e}+s_{j}\left(t\right)\right)\\ \tau_{i}\cdot v_{j}^{\prime } & =-v_{j}+f\left(a_{ie}\cdot u_{j}-a_{ii}\cdot v_{j}+a_{in}\cdot n_{j}-\theta_{i}\right)\\ \tau_{n}\cdot n_{j}^{\prime } & =-n_{j}+a_{n}\cdot u_{j}^{p}\left(1-n_{j}\right), \end{array}$$ (1)

with *j* ∈ {1, …, *N*}, where *N* is the number of interconnected populations, or *u* − *v* − *n* triplets, where each such triplet represents a tightly recurrently connected population of excitatory and inhibitory neurons with fast AMPA synapses (*u*_*j*_), a slow NMDA component of the excitatory synapse (*n*_*j*_), and slow GABA synapses (*v*_*j*_). *a*_*γδ*_, for *γ*, *δ* ∈ {*e*, *i*, *n*}, give the coupling strengths between different components of each population (these values do not vary between the populations in the model). ${\tilde{u}}_{j},\;{\tilde{v}}_{j},$ and ${\tilde{n}}_{j}$ represent coupled populations:

$${\tilde{\alpha }}_{j}=\left(\alpha_{j}+c_{z}\underset{k\neq j}{\sum }\alpha_{k}\right){\left(1+c_{z}\;\left(N-1\right)\right)}^{-1},$$ (2)

where *α* ∈ {*u*, *v*, *n*}, and *c*_*z*_ represents the relevant coupling from population *k* to population *j*: *c*_*e*_ for NMDA (*n*) and AMPA (*u*) synapses and *c*_*ei*_ for the inhibitory (*v*) synapses. The denominators ensure the excitation and inhibition remain bounded. Thus, *c*_*e*_ and *c*_*ei*_ give interpopulation coupling strengths, while *a*_*ee*_, *a*_*ei*_, *a*_*ie*_, and *a*_*ii*_ give intrapopulation coupling strengths. Fig 1 shows a schematic of the connectivities. The interpopulation excitatory coupling *c*_*e*_ represents overlap between populations. The mutual inhibition, *c*_*ei*_, allows for competitive dynamics between populations, as would the value of *c*_*ie*_, the interpopulation coupling strength from *u*_*j*_ to *v*_*k*_ (*j* ≠ *k*). However, we found that both of these connections provide similar dynamics to the results described in *Results* (see, e.g., Table 1 in the weak coupling case in S3 Text); thus, we have set *c*_*ie*_ = 0 for simplicity.

We assume coupled populations reside in neighboring areas, so that delays need not be considered, and that *τ*_*n*_ > *τ*_*i*_ > 1, where we have rescaled time so that the timescale is 1 for the fast excitation. An input stimulus, *s*_*j*_(*t*), allows the system to be activated. This stimulus is a square wave, for which we may adjust the amplitude, *A*_*j*_, as well as the width, *w*_*j*_, and onset time, *t*_0*j*_. The firing rate is given by $f\left(x\right)=\sqrt{\frac{x}{1-\text{exp}(-\beta \cdot x)}}$. We have chosen this firing rate since it gives an approximation of the firing rate of a noisy quadratic-integrate-and-fire spiking neuron [32, Chapter 10]. In S1 Text, we show an example simulation of the spiking model and compare it to the one-population version of the Wilson-Cowan equations.

Since we do not explicitly model membrane potential or spiking, there is no voltage dependence to the NMDA component; rather, it acts as a slow excitatory input. The NMDA component also saturates at 1, while the faster variables, *u* and *v*, are not constrained. The parameters *a*_*n*_ and *p* in the NMDA equation allow us to adjust the average NMDA level when that component is active; larger *p* also results in faster NMDA activation since *u* is typically larger than 1 when activated. Note that the behavior is fairly insensitive to *p*; the *n* − *u* null surface (where *n*′ = 0) is sigmoidal and *p* increases the gain of this sigmoid.

Unless otherwise specified, the parameters used are *N* = 5, *τ*_*i*_ = 12, *τ*_*n*_ = 144, *c*_*e*_ = 0.001, *c*_*ei*_ = 0.03, *a*_*ee*_ = 14, *a*_*ei*_ = 10, *a*_*en*_ = 4, *θ*_*e*_ = 6, *a*_*ie*_ = 20, *a*_*ii*_ = 8, *a*_*in*_ = 0.1, *θ*_*i*_ = 5, *a*_*n*_ = 2, *β* = 1, *p* = 2. These parameters allow for tristability among three behaviors of interest: a low nonzero steady state, a low-amplitude oscillation around this low steady state, and a large-amplitude oscillation. In our model, “active populations” refer to populations that are engaging in large-amplitude oscillations. Note that we vary *N*, *τ*_*i*_, *τ*_*n*_, *c*_*e*_, and *c*_*ei*_ to study their effects below. In particular, we find that there are open sets of these parameters that allow for the dynamics of interest, so that the precise values used are not critical to obtain the results shown.

Our protocol for simulating working memory is to load a memorandum via a square wave pulse stimulus (*s*_*j*_(*t*) in Eq (1)). Each stimulus is associated with the excitatory component of a specific population, so that a particular population may be selected for a memorandum. Multiple populations may be selected and provided stimuli either simultaneously or serially. Varying each of the amplitude *A*_*j*_, onset time *t*_0*j*_, and width *w*_*j*_ may produce different activation patterns, so that the observed activity is a direct result of the selected parameters and sequence of feed-forward inputs.

Each stimulus corresponds to a feature of an item present in the environment, so that persistent activation of a population selected by a stimulus corresponds to that feature’s representation within the neuronal network, where as mentioned above we allow for some overlap of excitatory populations, as represented by a nonzero *c*_*e*_ value. We then study how the system responds to patterns of stimuli, allowing their amplitudes, widths, and onset times to vary. We are particularly interested in phase synchrony and asynchrony of the large amplitude oscillations across the excitatory components of the populations. In our model, synchrony corresponds to different features that are associated with one another held active in working memory, while asynchrony corresponds to independent features held active. These behaviors correspond to binding and working memory capacity, respectively. We further study the existence and stability of these states while varying key parameters using XPP-AUTO [33].

However, since we obtain these network behaviors through a feedforward excitatory impulse to the excitatory components, we are further interested in which states are in fact accessible from some other given state. In systematically studying what patterns may be obtained from other patterns following a selective stimulus, the combinatorics involved quickly threaten to make the problem intractable. Thus, we limit our study to just two and three active populations, which we will refer to as diads and triads, respectively. We begin with either a diad or triad, and then use the protocol as described above, providing a single, selective stimulus of a chosen amplitude and width either to an active population or to an inactive one, and allow the network to evolve for some time afterwards. The resultant pattern is then determined to be accessible from the initial pattern. The outcome of stimulating the system is phase-dependent, as is the case in many oscillatory systems. Since we are interested in behaviors that may be more robust, we narrow our results to only include those that contain multiple-millisecond intervals of time that produce the same resultant activity for a stimulus of fixed width and amplitude. All of the included results may be obtained with a stimulus of fixed width and amplitude over intervals no smaller than 6ms, consistent with intervals associated with gamma frequency phase locking that some pyramidal neurons and interneurons display, as described in Senior et al. [34] They found that these neurons locked their firing to two phases within one gamma (30–80Hz) period. Thus, the smallest interval of time that these neurons might be able to distinguish is 6–7ms. Additionally, synchronization between neurons has been shown to occur within a time window of around 10ms [35]. For simplicity, we only consider a network with *N* = 5 when we explore the accessibility of operations involving diads and triads.

## Results

The model produces persistent elevated firing states (above baseline levels) in response to inputs to selected populations in networks consisting of multiple populations, consistent with what has been observed in neurophysiological studies (e.g., [5]). This persistent working memory activation may occur either as steady-state or oscillatory firing rates, depending on the value of the relative speed of the inhibitory synapses. For the case of oscillatory dynamics, analogues of several critical features of working memory arise naturally and robustly, which, in addition to the persistent elevated activity, include working memory capacity and binding. In contrast, for the steady-state case, it is difficult to obtain multiple populations active at once, and in the case of uniform connectivity, the activity would be indistinguishable (i.e., they would all be active with the same firing rate). An analysis and description of the mechanisms underlying the oscillatory behavior of an isolated *u* − *v* − *n* triplet is presented in S2 Text.

We found that our model allows for stable oscillatory states involving a single population (SO: Single Oscillator) or multiple coupled populations. In the latter case, three basic types of oscillations may occur: out-of-phase (OP), synchronous (S), and mixed phase (MP). By OP, we mean that if there are *k* active populations, then each of the *k* occupy separate parts of a cycle. For example, if *k* = 2, then the two populations oscillate a half cycle apart, and if *k* = 3, each population oscillates a third of a cycle apart. In our model, OP populations represent single-feature, distinct items in memory. S means all populations fire in-phase with one another and represent bound items in memory, while MP means that some populations are synchronized and others are out of phase, corresponding to bound and distinct items in memory, respectively. For example, it could be the case with three populations that two are synchronized while the third fires out of phase, corresponding to two distinct objects held in memory: one with two features and one with a single feature.

Although all of the network’s parameters play a part in the allowed dynamics, we observed that different parameters played roles of varying importance to each of the states. We thus found it fruitful to first explore some of the features and constraints of the S and especially the OP states individually, and then to see how these attracting states naturally combine to form more complex MP activity that may represent multiple rich, multi-feature memories.

### Organization of results

In Out-of-phase oscillations and distinct memoranda we look at OP oscillations in isolation. We look at both example simulations (*OP dynamics*) and some of the central results on constraints on the number of populations that may oscillate OP (*OP oscillations and working memory capacity*).

In *Synchronous oscillations and binding*, we turn our attention to S oscillations. Again, we examine example dynamics (*S dynamics*) and limits on how many populations may oscillate synchronously (*Maximum S populations*).

In *Mixed-phase oscillations: Synchronous and out-of-phase*, we see how OP and S oscillations may both occur and can lead to MP oscillations, which are a central feature of the model. We look at example activities (*MP dynamics*) and then try to gain insight into how we might attain the different oscillatory modes by looking at two different simplifying assumptions. First we see how the coupling strengths allow for the different states in the reduced case of just 2 coupled populations (*Two populations: Effects of coupling strengths*), and then turn to the case of weak coupling (*Weak coupling*) and examine the basins of attraction for S, OP, and MP modes with just 3 populations.

Finally, in *Biological considerations and applications*, we examine the model in relation to biological and cognitive considerations. We first focus on one of the main features of the model by exploring how the network can rapidly form and transition between a rich variety of MP relationships in the context of binding (*Feature binding* and *Variable binding*). We then more carefully examine the transitions the model network may make (*Accessible operations*), and compare some of the frequency relationships in the model to experimental results (*Frequencies*).

### Out-of-phase oscillations and distinct memoranda

**Fig 2:**
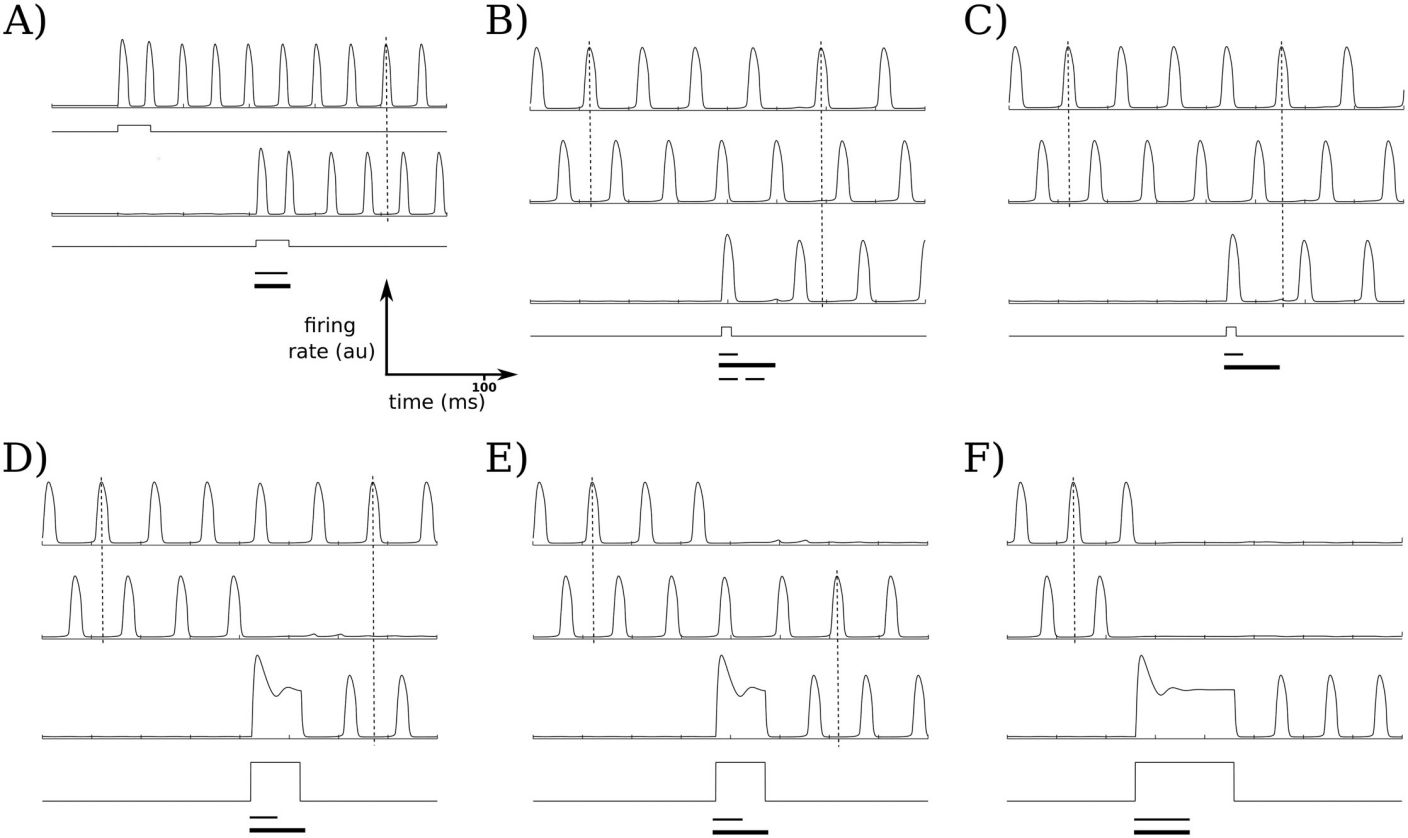
OP dynamics for up to 3 active populations. For a network size of 5 populations (*N* = 5), all combinatorial possibilities for up to 3 active OP populations may be realized. Square waves in these and similar figures indicate the stimuli given to the population just above the given wave, and the vertical dashed lines in each plot allow for phase comparisons across different active populations. The first bar below each group of traces shows the interval of starting times in which the same stimulus (fixed amplitude and width) will produce the same result; the second bar shows the length of the period of the oscillation that is active before the stimulus is applied (note: the period may change after the stimulus is applied); the third bar in (B) is explained below. (A) The network starts at a nonzero, nonactive baseline firing rate. The first stimulus selectively activates the first population, while the other populations remain inactive with low firing rates. A second population is then activated; for these parameters and stimulus strength, almost any stimulus onset time will induce the OP state with 2 active populations, as the bars show. (B, C) Stimulating a third population with a short stimulus induces the OP state with 3 active populations. Either activation ordering may occur, depending on the phase the stimulus is presented; (B) and (C) show the two different orderings. The third, dashed, bar below the stimulus trace in (B) shows the interval of onset times that induce the OP state with three active populations; the first interval shows the onset times that produce (B) while the second interval shows the onset times that produce (C). (D, E) Larger and wider stimuli may deactivate either of the active populations, so that the network remains in the OP state but with different active populations (WTS scenario). (F) Maintaining the amplitude of the stimulus from the WTS case but increasing the stimulus width allows the third selected population to deactivate both active populations and become the only active population (WTA scenario).

OP oscillations of the interacting populations in the network (Fig 2) display the fundamental signature of working memory in the model. Each distinct memorandum being held active is associated with a different phase, similar to what has been proposed in previous work (e.g., [27, 36–38]). In contrast to some of this previous work, however, the present firing rate networks are self-contained. That is, they do not explicitly receive any external signal to organize their relative phase timings. Instead, mutual inhibition allows for competition between the populations so that only one population (or group of synchronously firing populations as discussed in *MP dynamics*) is active during any given portion of a cycle. Thus, for a given cycle duration (determined by conditions discussed in detail in *OP oscillations and working memory capacity* and *Two populations: Effects of coupling strengths*), there is a limit to the number of OP populations that the network may support. In the model this corresponds to working memory capacity. We will also refer to this capacity as the network capacity, as it is a fundamental property of the networked populations.

#### OP dynamics

Depending on the timing and strength of the stimulus given to each population, a selected population may either oscillate out-of-phase with currently active populations in the network cycle, or it may compete with one or more populations, quenching their activity while remaining active itself. More concretely, consider the case of a 5-population network (*N* = 5) with a network capacity of 3 active OP populations. If no input is given, all of the populations fire at low, but non-zero, rates. Selectively stimulating first one of the populations, and at a later time a second population, allows for these two populations to be simultaneously active, exhibiting OP oscillations as shown in Fig 2A. The result of subsequently selectively stimulating a third population depends on the strength, width, and onset time of the stimulus. Stimuli of sufficient width and amplitude can result in a winner-take-all (WTA) scenario (Fig 2F), where in the 3-population-capacity network the activation of the third population suppresses the first two populations sufficiently to become the only active population. Weaker stimuli allow for a winner-take-some (WTS) scenario (Fig 2D and 2E); in this case, the third population quenches one population (whose activity returns to baseline level) and becomes persistently active, leaving two OP populations. Both of these cases could represent selective forgetting due to interference. For example, they could correspond to situations in which attention is shifted to one item at the expense of other attended memoranda, effectively resulting in the forgetting of those items that had been held active in working memory.

If the stimulus is of moderate strength (that is, of sufficient amplitude and duration to activate the population, but not sufficient to quench another active population), then, depending on the onset time of the stimulus, the selected population may become interleaved with the other active populations in the current cycle (Fig 2B and 2C). In this case, all populations may fire OP, with the ordering of the newly activated population with respect to the already-active populations within the cycle determined by the stimulus onset time (e.g., Fig 2B vs 2C).

#### OP oscillations and working memory capacity

As mentioned above, for any set of nontrivial parameters (e.g., *c*_*ei*_ ≥ *ϵ* > 0) there is a maximum number of OP populations that may be active, resulting in a finite network capacity.

We note that a working memory capacity is entailed either by nonzero coupling (i.e., one of *c*_*e*_, *c*_*ei*_, *c*_*ie*_ is positive), or by the temporal resolution of the network. That is, in a biological network, we would not expect perfect synchronization to occur with any regularity; rather, the detection of synchrony by the network would need to operate within certain temporal bounds, so that oscillations that are within some threshold time measure of being synchronous are in fact read out as synchronous. Indeed, the same is true numerically in our model simulations, if for very narrow temporal bounds. In either case, there is a minimum distance, say *η* > 0, that the excitatory peaks, e.g., may be from each other (in the first case, the minimum distance may arise due to the attracting or repelling basins of the synchronous state that result from nonzero coupling strengths, for example). Thus, if *T* is the period of the network oscillation, the maximum working capacity would be $\frac{T}{\eta }$, which is finite for any fixed set of parameters.

Both the mutual inhibition, *c*_*ei*_, and the timescales, *τ*_*i*_ and *τ*_*n*_, are critical in determining this capacity. We expect the value of *c*_*ei*_ to play a strong role in determining the network capacity since the OP dynamics fundamentally arise from mutual inhibition between the populations in the network (see Methods and *Two populations: Effects of coupling strengths*). The effect the timescales have on the capacity is somewhat more nuanced. The timescale of inhibition works differentially on the excitatory populations, depending on their phases. Briefly, we find that, in particular, larger values of *τ*_*i*_ increase the time the excitatory populations spend near zero more than the time they spend away from zero, providing a greater window of opportunity for other populations to be active. We now look at this in greater detail.

We observe that there appear to be two qualitatively different features to the excitatory oscillation: (1) a pulse-like portion, where the activity rapidly increases and rapidly decreases, which we will call the “active phase”; (2) a portion where the firing rate stays very close to zero, which we will call the “quiescent phase”. Although the division between these phases is necessarily arbitrary, a natural threshold choice is the low fixed point, u_*_ (the baseline firing rate). Thus, for some fixed *j*, the active phase of a given oscillation, *T*_*a*_, is defined as the interval of time such that *u*_*j*_ > u_*_, while the quiescent phase of an oscillation, *T*_*q*_, is the interval of time such that *u*_*j*_ ≤ u_*_. Thus, *T*_*a*_ is a measure of the width of the pulse, while *T*_*q*_ is a measure of the time it spends near zero. Note that *T*_*a*_ + *T*_*q*_ = *T*, the period of the oscillation. *T*_*a*_ is mostly determined by the rise time of the inhibition, which is strongly affected by the excitatory population. In contrast, *T*_*q*_ is mostly determined by the decay time of the inhibition. See S2 Text for more detail. Thus, while both *T*_*a*_ and *T*_*q*_ increase as *τ*_*i*_ increases, we expect $\frac{T_{q}}{T_{a}}$ to increase as well. Indeed we find this to be the case; for example, with our original parameters (including *τ*_*i*_ = 12) and one active population, *T* ≈ 50ms, and $\frac{T_{q}}{T_{a}}\approx \frac{28}{22}\approx 1.3$. If we increase the timescale of inhibition up to *τ*_*i*_ = 20, *T* ≈ 76ms, and $\frac{T_{q}}{T_{a}}\approx \frac{48}{29}\approx 1.7$. Thus, the ratio $\frac{T_{q}}{T_{a}}$ has increased by a factor of about 1.3. We therefore might intuit that more populations can be active OP as *τ*_*i*_ increases, since more pulses from other populations may “fit” between the pulses of an already-active population. That is, since there is a proportionally larger interval of time the population spends near zero than the interval of time it is active, there is greater opportunity for other populations to be active during this quiescent phase. We also note that while it is important for *τ*_*n*_ to be large enough so that the NMDA population may reactivate the excitatory population (see S2 Text), it otherwise does not affect the period of the oscillation very much. For example, with our original parameters (including *τ*_*n*_ = 144) and one active population, recall that *T* ≈ 50ms; increasing *τ*_*n*_ to 240 slightly *decreases* the period to *T* ≈ 49ms. (We note that it is not surprising for *τ*_*n*_ to have an inverse relationship with the period, since *τ*_*n*_ mostly controls the decay time of the NMDA; thus, *n* stays slightly higher for larger *τ*_*n*_, resulting in slightly faster activation times for *u* and *v*. Since *τ*_*n*_ has little effect on the decay time of inhibition, which is mostly determined by the time constant *τ*_*i*_, we see that increasing *τ*_*n*_ may in fact decrease the period).

**Fig 3:**
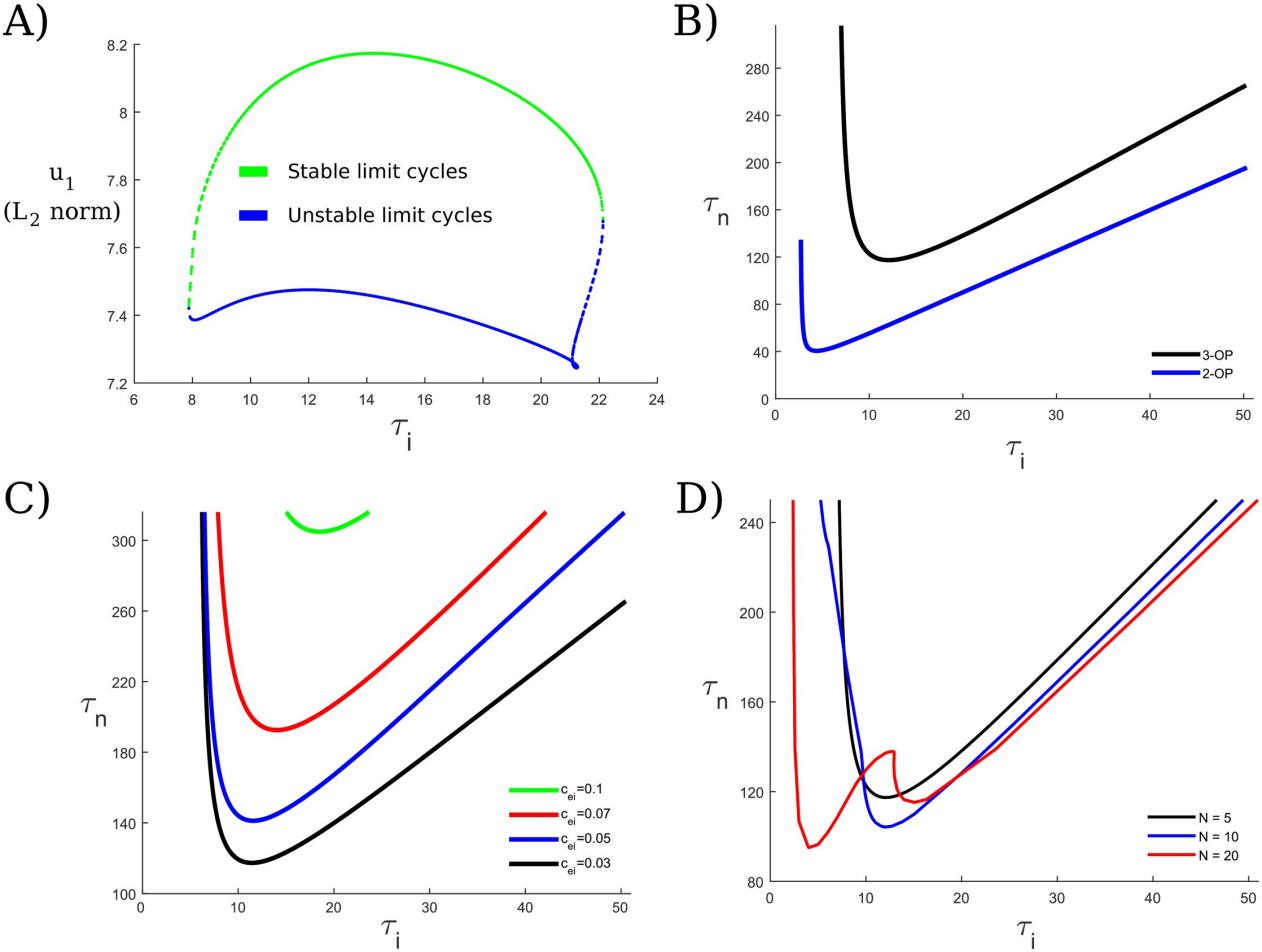
Stability of OP oscillations and working memory capacity. For fixed *τ*_*n*_, there is a window of *τ*_*i*_ values for which oscillations exist. We examine the existence and stability of the active out-of-phase populations, corresponding to distinct, single-featured memoranda. (A) Here, we look at the 3-OP state for a network of *N* = 5 populations, with *τ*_*n*_ fixed at 144 and the mutual inhibition *c*_*ei*_ fixed at 0.03. For *τ*_*i*_ too large or too small, the oscillations are lost as folds of limit cycles (for larger *N*, they may also be lost through torus or period-doubling bifurcations). (B) By following the limit points in (A) and keeping *N* fixed at 5, we may examine the dependence of the oscillatory states on both timescales, *τ*_*n*_ and *τ*_*i*_, for different OP states. Thus, we see how the capacity of the system depends on the timescales. Each curve is a curve of the limit points as shown in (A). Thus, the OP state with 2 active populations exists stably above the blue curve, and the 3-OP state exists stably above the black curve. (C) We may further examine how the capacity is affected by the strength of the mutual inhibition, *c*_*ei*_. Here, the 3-OP state exists above each curve for different *c*_*ei*_ values as indicated. As the mutual inhibition increases, the minimum *τ*_*n*_ value that supports the 3-OP state increases. Thus, we would like to keep the mutual inhibition low enough to support the 3-OP state within physiologically realistic synaptic timescales, but high enough to allow for the WTA state. (D) If we fix *c*_*ei*_ = 0.03, we may further explore how the network size *N* affects the 3-OP state. Overall, as *N* increases, the set of timescales that supports the 3-OP state does not change very much, generally increasing slightly. The bifurcation structure for *N* = 20 changes somewhat as well, so that the 3-OP state may destabilize through a torus or period-doubling bifurcation for lower *τ*_*i*_ values as well.

We may follow the oscillatory solutions in AUTO to determine exactly how the network capacity depends on the mutual inhibition and the system’s timescales. The OP states are lost as folds of limit cycles or destabilize via torus or period-doubling bifurcations as *τ*_*i*_ increases or decreases with *τ*_*n*_ fixed (Fig 3A illustrates this for the 3-OP state). In particular, we found that for *N* = 5 or 10, the solutions were lost as folds of limit cycles as in Fig 3A, while for *N* = 20 the oscillations sometimes first lost stability via the torus or period-doubling bifurcations (see Fig 3D). For *N* = 5 or 10, the 1-OP state (i.e., just one active oscillating population) is also lost as a fold of limit cycles (in *τ*_*i*_), suggesting we may gain intuition into why the *M*-OP states exist for certain *τ*_*i*_ and *τ*_*n*_ values for *M* > 1 by examining the 1-OP case in more detail, which we do at the end of S2 Text by examining how the separation of timescales allows for the existence of these solutions (i.e., why the ratio $\frac{\tau_{i}}{\tau_{n}}$ cannot be too large or too small). While there are certain trends in the behaviors of the solutions, we did not observe any significant changes in the dynamics near bifurcation. In the case of the inhibition, as *τ*_*i*_ increases, the period increases (as discussed at the beginning of this section), more than doubling (from 50 to 117 ms), and the maxima of the excitatory populations increase by about 25%, while the minima of the NMDA populations decrease by ≈33% (note that the NMDA generally peaks at or near saturation at 1, so this does not tend to change as we adjust parameters) and the maxima of the inhibitory populations decrease only very slightly, staying virtually constant (a change of ≈ 5%). We note that not all of these behaviors (or those described below for the case of varying *τ*_*n*_) are strictly monotonic, but rather they indicate the trends. For example, the amplitude of the inhibition increases very slightly with *τ*_*i*_ before it decreases; however, up to two significant figures it remains at 6.8. If *τ*_*i*_ increases beyond the high fold, the solutions tend to change from *M*-OP to (*M* − 1)-OP; e.g., 3-OP solutions are lost to 2-OP solutions. If *τ*_*i*_ decreases beyond the low fold, we get slightly different behavior. In this case, the solutions tend to change from *M*-OP to *M*-MP; i.e., one of the populations will synchronize with another one. In both cases, we see that the number of mutually OP groups decreases from *M* to *M* − 1.

For NMDA, as *τ*_*n*_ increases, the period decreases (as discussed at the beginning of this section) from 77 to 63 ms, the maxima of the excitation and inhibition stay virtually constant (changing by less than 1%) and the minima of the NMDA populations increase by ≈50% (since, of course, *τ*_*n*_ increases). As we can see from Fig 3D, there is a fold of limit cycles (for *N* = 5 and *N* = 10) for low *τ*_*n*_; increasing *τ*_*n*_ does not appear to cause the loss of existence or stability of the solutions. This is as expected, since in the limit *τ*_*n*_ → ∞, NMDA will simply stay high (once activated) as it acts essentially as a parameter. Thus, as we explore in more detail in S2 Text, the NMDA will always outlast the downstroke of the inhibition, allowing the excitation to reactivate. If we decrease *τ*_*n*_ to below the fold, we again can lose the *M*-OP state to the *M*-MP state, as one of the active populations will tend to synchronize with another one.

By following the above bifurcations, we may partition *τ*_*i*_ − *τ*_*n*_ space based on how many OP populations can be active for fixed *c*_*ei*_ (Fig 3B), or on the maximum number of OP populations that may be active for different values of *c*_*ei*_ (Fig 3C). In Fig 3 we see that we have open sets of *τ*_*i*_ and *τ*_*n*_ values that lie within physiological ranges where the network capacity is 3, matching experimental ranges of a capacity of 3–5 items in working memory. As we increase the number of populations, the capacity of the system remains more or less the same (Fig 3D), suggesting the capacity is only weakly dependent on the network size.

### Synchronous oscillations and binding

Different types of binding occur within the context of working memory. In all examples of binding, temporal synchronization of firing may play a significant role as the underlying mechanism. There are several different ways in which our model may achieve such synchrony between different populations in the network.

#### S dynamics

**Fig 4:**
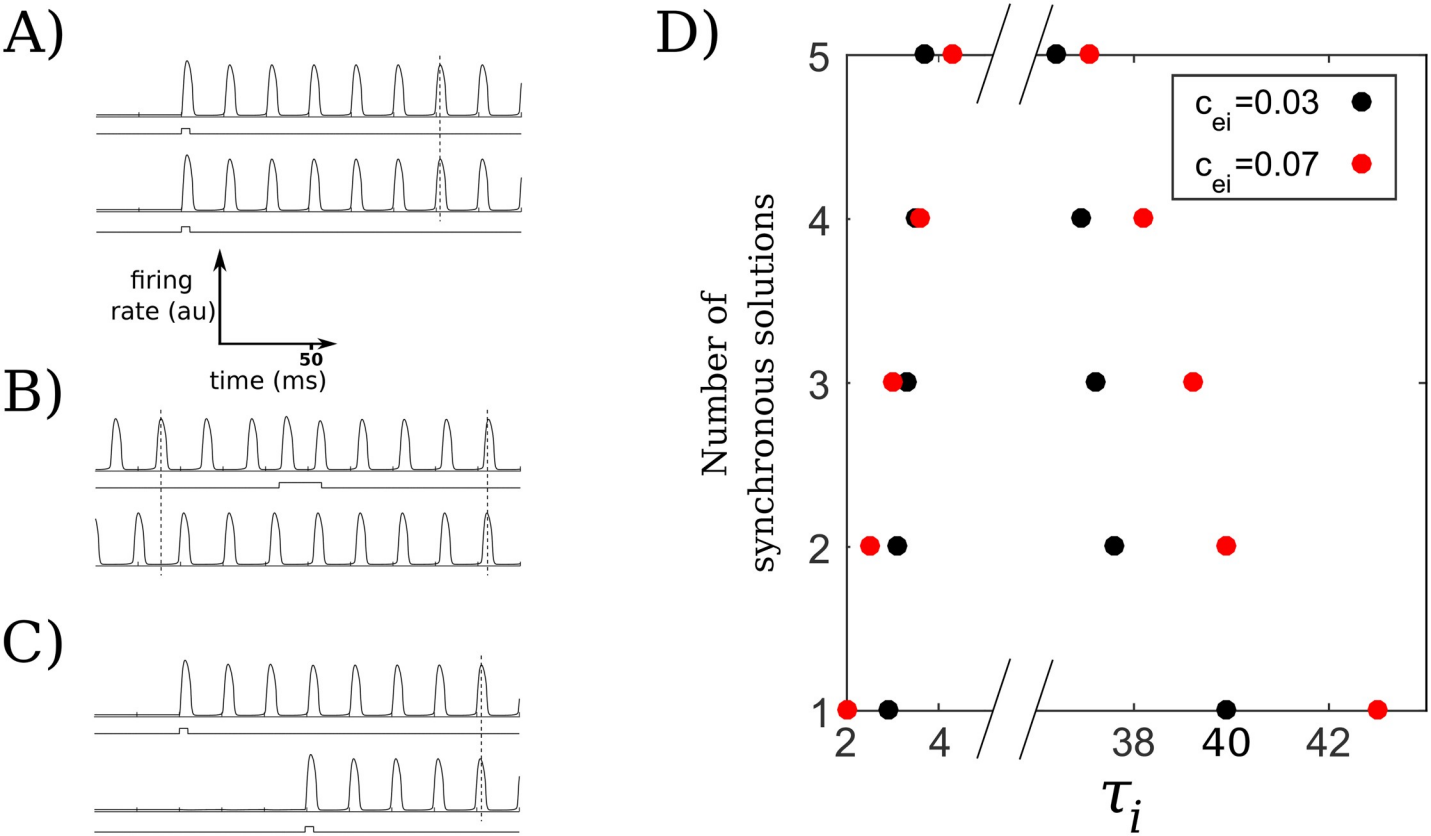
S dynamics and capacity. (A) Simultaneous stimuli can cause the selected populations to exhibit S oscillations. (B) If two populations are pairwise OP, selectively stimulating one can alter its relationship so that the two populations subsequently oscillate pairwise S. (C) Sequential stimuli of the right timing may also cause the selected populations to dynamically bind and oscillate S. (D) Synchronous capacity as a function of *τ*_*i*_ and *c*_*ei*_. For *M* = 1, …, *N* (here *N* = 5), the *M*-S solution is stable for all *τ*_*i*_ values between each respective pair of (same-colored) points. For example, the 5-S solution with *c*_*ei*_ = 0.03 is stable for 3.7 < *τ*_*i*_ < 36.4. The synchronous solutions are not stable outside of these intervals and are lost to different solutions, as indicated in the text.

In Fig 4, we see three such possible paths based on the pattern of inputs. Unsurprisingly, if two inactive populations, say populations 1 and 2 without loss of generality, are stimulated with similar stimulus parameters (onset time, amplitude, and duration), they will oscillate S (Fig 4A). If two populations are active OP, we may apply a stimulus to either one so that they synchronize (Fig 4B). Finally, if population 1, for example, is oscillating in isolation, population 2 may also synchronize with population 1 if given a small stimulus within a certain time window of the phase of population 1 (in particular, close to when the excitatory component has an upstroke) (Fig 4C). The latter two cases illustrate the attractive nature of S oscillations in the model, which we further confirm through numerical continuation. We note there are other ways in which populations may pairwise oscillate S in the network, some of which are explored in *Mixed-phase oscillations: Synchronous and out-of-phase* and *Biological considerations and applications*.

#### Maximum S populations

We first observe from Eq (1) that if all *N* populations are active S in a given network, they oscillate as a single population with inhibition given by *a*_*ei*_. Thus, we might expect the maximum number of populations that can oscillate synchronously to depend more on the intrapopulation parameters, such as *a*_*ei*_. Indeed, this solution is eventually lost as a fold of limit cycles as *a*_*ei*_ increases. However, the interpopulation coupling values, such as *c*_*ei*_, do affect the stability of this oscillating solution. We have observed these solutions to be lost as branch points (with no stable alternate branch) or period doubling bifurcations. Although the types of bifurcation may change, the types of changes the parameters cause match our expectations: As *c*_*ei*_ increases, the *a*_*ei*_ value at which the solution destabilizes decreases. Increasing the *c*_*e*_ value correspondingly increases the *a*_*ei*_ value at which the bifurcation occurs. Furthermore, decreasing the number of active populations increases the *a*_*ei*_ values at which the stable oscillations are lost, so that, for example, with the same intrapopulation and interpopulation coupling strengths, 4 S populations remain stable for higher *a*_*ei*_ values than 5 S populations do.

For other parameters, the behavior may be somewhat more complicated. For example, consider the range of *τ*_*i*_ values that allows for the stable existence of *M*-S solutions for *M* ∈ {1, … *N*} (of course the 1-S oscillation is just a single oscillator, SO). As we see in Fig 4D, as *M* increases from 1 to 5 with *c*_*ei*_ fixed at 0.03 or 0.07, the interval of *τ*_*i*_ that allows for the stable existence of the *M*-S oscillations narrows monotonically, resulting in a nest of intervals, each of which is strictly contained in the interval below it. However, the result of increasing *c*_*ei*_ from 0.03 to 0.07 varies somewhat on the number of synchronous oscillating populations. The right boundary, corresponding to the largest *τ*_*i*_ that supports the synchronous solution, increases with increasing *c*_*ei*_, especially for fewer oscillating populations. The left boundary, corresponding to the smallest *τ*_*i*_ that supports the synchronous solution, increases for larger *M* (*M* = 4, 5) and decreases for smaller *M* (*M* = 1, 2, 3). As mentioned above, when *M* = *N*, the whole network oscillates as a single group, as can be seen with the appropriate substitutions in Eq (1); thus, while varying *c*_*ei*_ does change the stability of this oscillation, as can be seen for *M* = 5 in Fig 4D, the other characteristics, such as the amplitudes and period of oscillation, do not change at all. However, these can and do change for *M* < *N*; in particular, increasing *c*_*ei*_ tends to increase the amplitudes of the excitatory and inhibitory populations. We explore these changes in dynamics and explore the changes we see in Fig 4D in more detail in S4 Text.

### Mixed-phase oscillations: Synchronous and out-of-phase

We have seen above in *Out-of-phase oscillations and distinct memoranda* and *Synchronous oscillations and binding* that the network allows for both S and OP oscillatory states as attractors. When such states coexist in the network (i.e., at least two populations are pairwise OP and at least two populations are pairwise S), we say the populations oscillate MP (mixed-phase). An important result in our current work is that the model network can support a large number of attracting MP states, where the pairwise OP populations correspond to distinct items in memory and the pairwise S populations correspond to bound features of a memory.

Thus, multiple items in memory, each with multiple features, can be represented within our framework. Indeed, we found a range of combinatorial arrangements and dynamics emerge in the model network that are rich enough to map onto or underlie many of the aspects of binding. We explore some of these dynamics below, and other examples that may be relevant to feature and variable binding in *Feature binding* and *Variable binding*.

#### MP dynamics

When a quiescent or active population is stimulated, it may oscillate pairwise OP or S with currently active populations. Here we see three ways whereby we can obtain MP oscillations with a selective stimulus, so that a feature is added to an item in memory.

**Fig 5:**
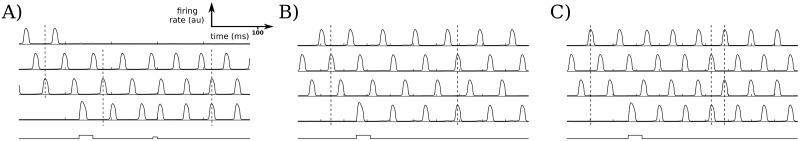
MP dynamics. (A) Stimulating an inactive population when the network is already at capacity (3 OP populations here) may cause one of the active populations to become quiescent, even without a strong stimulus. A subsequent stimulus produces similar behavior as in Fig 4B, so that stimulating an active population may allow it to change its relationship from pairwise OP with both other active populations to oscillate S with one of the two other active populations, and OP with the second. (B–C) Stimulating a quiescent population can cause various dynamic bindings and MP dynamics. For example, changing only the timing of the stimulus can alter the resulting patterns of synchronization. In (B), the stimulated population synchronizes with one of the three OP populations. (C) Adjusting the onset time of the stimulus may result in additional interactions, so that the network transitions from 3 OP populations to 2 OP pairs, where both populations in each pair oscillate S.

In Fig 5A–5C, there are initially 3 active OP populations (say populations 1–3). If population 3, for example, receives a stimulus, then its timing may be readjusted to synchronize with population 2. Alternatively, a quiescent population (say population 4) may be stimulated. Since the capacity of the network is reached with 3 OP populations (or really, 3 groups of mutually OP populations; that is, populations within each group oscillate S and populations in different groups oscillate mutually OP), in order for population 4 to remain active, either at least one of populations 1–3 must be quenched or at least two of populations 1–4 must synchronize. In Fig 5A, activating population 4 with a somewhat weaker stimulus quenches the activity of population 1 as might occur in forgetting, suggesting independently of the numerical analysis in *OP oscillations and working memory capacity* that the capacity of the network is indeed 3. Further stimulating population 4 causes it to synchronize with population 2, in a similar way that occurs in Fig 4C when sequential stimuli cause two populations to oscillate S. However, since all of the populations are coupled to one another, more complicated effects may occur as well. For example, in Fig 5C, once population 4 is stimulated, it also synchronizes with population 2 as in Fig 5B; however, populations 1 and 3 are perturbed enough to synchronize as well. We note that the only differences between Fig 5B and 5C are the onset times of the stimulus to population 4. More generally, the stimulus parameters determine which populations oscillate S and which do not. We also see that in both Fig 5B and 5C four populations are active following the stimulus. Thus, the number of active populations depends in part on how many OP populations may be active (see *OP oscillations and working memory capacity*) and in part on how many S populations may be active (see *Maximum S populations*). We consider additional exmaples of MP states in *Feature binding* and *Variable binding*.

#### Two populations: Effects of coupling strengths

**Fig 6:**
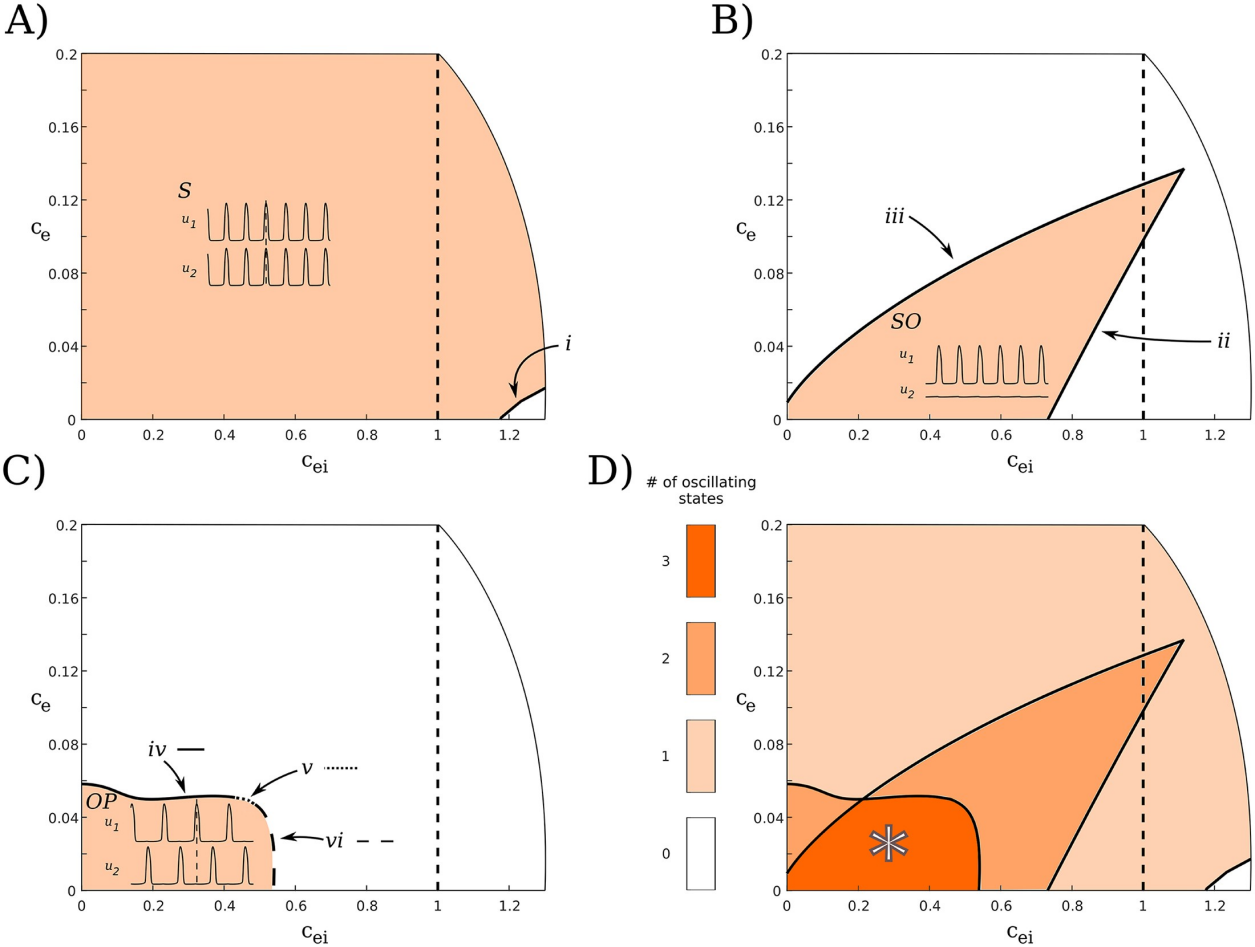
Stability as a function of coupling strengths for *N* = 2. The dashed line at *c*_*ei*_ = 1 indicates the interpopulation and intrapopulation inhibition are the same. All of the dynamics of interest exist stably when the interpopulation coupling is less than the intrapopulation coupling (to the left of the dashed line), as desired. (A–C) Colored regions indicate areas that the oscillatory states shown stably exist. Roman numerals refer to the boundary curves of the regions as indicated. (A) The S state exists to the left of (i), which is a curve of branch points of limit cycles (BPLCs). (B) The SO state exists to the left of (ii), a curve of folds of limit cycles (FLCs) (these folds are the only ones in this diagram that arise from a subcritical Hopf bifurcation) and to the right of (iii), another curve of FLCs. (C) The OP state exists below (iv), a curve of FLCs, and (v), a curve of torus bifurcations, and to the left of (vi), a curve of BPLCs. The line style for each curve is shown to the right of each roman numeral for clarity. (D) All of the regions in (A–C) superimposed. (*) indicates the region of interest, where all three oscillating states (S, SO, and OP) exist stably.

Having seen that our model supports both OP and S dynamics that naturally combine to allow for richer MP dynamics, we look at the reduced case of two populations to distinguish how the interpopulation coupling affects the existence of the OP and S states, as well as the case where only a single oscillator (SO) is active. We note that the stable existence of the SO state is necessary for WTA dynamics; however, the SO state may also stably exist with lower coupling values that do not allow for WTA, and so it is not sufficient for WTA dynamics. The coupling strengths are crucial in being able to maintain any of these oscillatory dynamics. We see that in the coupling-strength space in Fig 6D, only the region * supports all three oscillatory states. Both SO (Fig 6B) and OP (Fig 6C) states exist stably in bounded sets, so that if either *c*_*e*_ or *c*_*ei*_ values become too large, the states will be lost. In contrast, there is no indication that the S state only exists in a bounded region (Fig 6A). If *c*_*ei*_ values are large enough with *c*_*e*_ small, the S state may lose stability (to the right of (i) in Fig 6A). However, we see from Eq (2) that as *c*_*e*_ → ∞, each excitatory population only gets excitation from the complementary population, so we expect synchrony to continue to stably exist for arbitrarily large *c*_*e*_ values. All three oscillatory states appear to exist stably when either *c*_*e*_ or *c*_*ei*_ is zero and as the other value approaches zero (i.e., along one of the axes near the origin), suggesting that weak coupling is sufficient for these states to exist. In S3 Text, we explore the attracting states in the weak coupling limit.

Although the general features described above for Fig 6 match our intuitions, some of the particular features are unforeseen and may warrant further scrutiny. We now look more carefully at the regions that the S, SO, and OP states stably exist in coupling-strength space in turn, describing the curves of bifurcations and the behavior of the oscillations with changing coupling strengths.

The S state (Fig 6A) exists for all relevant coupling strengths. Neither the period nor the amplitude of the oscillations change as *c*_*e*_ and *c*_*ei*_ change. However, if *c*_*ei*_ increases beyond (i), a curve of branch points of limit cycles (BPLCs), only the low and high steady states are stable.

The SO state (Fig 6B) lies to the right of (iii), a curve of folds of limit cycles (FLCs). As we can see in Fig 6D, the oscillatory states that are available for lower *c*_*ei*_, to the left of (iii), are either S or, for sufficiently small *c*_*e*_ values, OP. The SO state is bounded on the left by (ii). Curve (ii) is also a curve of FLCs; however, unlike the other such curves in Fig 6, these folds arise from a subcritical Hopf bifurcation.

For fixed *c*_*e*_ and increasing *c*_*ei*_, the SO solutions show increases in the period (from 48 to 74 ms) and the maxima of both the excitatory (increasing by ≈ 20%) and inhibitory (≈ 40%) oscillations. These changes continue until the SO state is lost to a high fixed point. As we might expect from a subcritical Hopf (in particular, one that involves a fixed point away from zero), this change happens rapidly, with no changes before bifurcation suggestive of the loss of stability of the solution. For fixed *c*_*ei*_ and increasing *c*_*e*_, the SO solutions show slight decreases in the period (from 53 to 51 ms) and the maxima of the excitatory and inhibitory populations (both decrease by ≈ 12% or less).

We note that once *c*_*ei*_ increases beyond the subcritical Hopf bifurcation (curve (ii)), the system displays steady state bistability between a down state and an up state with the same inhibitory timescale as for the case of oscillations. However, a much larger input is required to switch between active populations in this case. For example, suppose *c*_*e*_ = 0.001 and population 1 is active. If *c*_*ei*_ = 0.7 (“after” the Hopf, so that population 1 has a large-amplitude oscillation), stimulating population 2 with a width of 50ms and an amplitude of 3 (arbitrary units) is sufficient to allow population 2 to become active and quench the activity of population 1. However, if *c*_*ei*_ = 0.8 (“before” the Hopf, so that population 1 is in the up state), a stimulus to population 2 with the same width requires an amplitude of greater than 36 in order for population 2 to activate, sending population 1 back to the down state. This up state is only stable for smaller *c*_*e*_ values; for larger *c*_*e*_ the only stable state of the system is S activity. We see in Fig 6D that the S state exists to the right of (ii) for low *c*_*e*_ values as well.

The region of definition of the OP state is more complicated. It is defined by (iv), a curve of FLCs, (v), a curve of torus bifurcations, and (vi), a curve of BPLCs. These curves do not decrease in *c*_*e*_ monotonically as *c*_*ei*_ increases. We see that that there is a small interval of *c*_*e*_ values around 0.06 where the OP state exists stably for small *c*_*ei*_ values, but is lost for higher values (e.g., *c*_*ei*_ not quite 0.2). Surprisingly, once the OP state is lost here, we initially can only get the S state. As *c*_*ei*_ increases further, we may also obtain the SO state. Perhaps the most unanticipated feature of the OP region occurs near the local minimum of (iv). There is a very small *c*_*e*_ interval, near *c*_*e*_ = 0.05, where the OP state is lost (to a region where S and, for larger *c*_*ei*_, SO both exist, as seen in Fig 6D) and then regained as *c*_*ei*_ increases, before finally being lost again for large *c*_*ei*_. We explore the behaviors of the OP state further in S4 Text, including changes in the dynamics that occur with increasing *c*_*e*_ or *c*_*ei*_ that may lead to the bifurcations we observe.

#### Weak coupling

Surprisingly, both S and OP oscillations are stable as the coupling strengths *c*_*e*_ and *c*_*ei*_ approach 0 with *N* = 2, as we saw above in Fig 6. Although we have chosen coupling strengths that allow for stronger interactions between populations (allowing for, e.g., WTA and WTS dynamics), we may gain some insight into our system by examining the limit of weak coupling. In this case we first assume that the populations are oscillating.

In S3 Text, we outline how we may use weak coupling theory to reduce our system of *N* coupled populations from a (3 ⋅ *N*)-dimensional system to an (*N* − 1)-dimensional system involving the phase differences. Briefly, we restrict our attention to weakly-coupled oscillatory populations. Since each 3-dimensional population (involving *u*, *v*, and *n* variables) is assumed to be on a limit cycle, its state can be referenced by its phase on that limit cycle, *θ*_*j*_, where *j* is the population index. We can further reduce the number of dimensions by one by referencing each population’s phase relative to *θ*_1_, the first population’s phase. Thus, we are left with *phase differences* *ψ*_*j*_. Since *ψ*_1_ ≡ 0, we can examine the attractor structure of three weakly-coupled populations in the plane.

**Fig 7:**
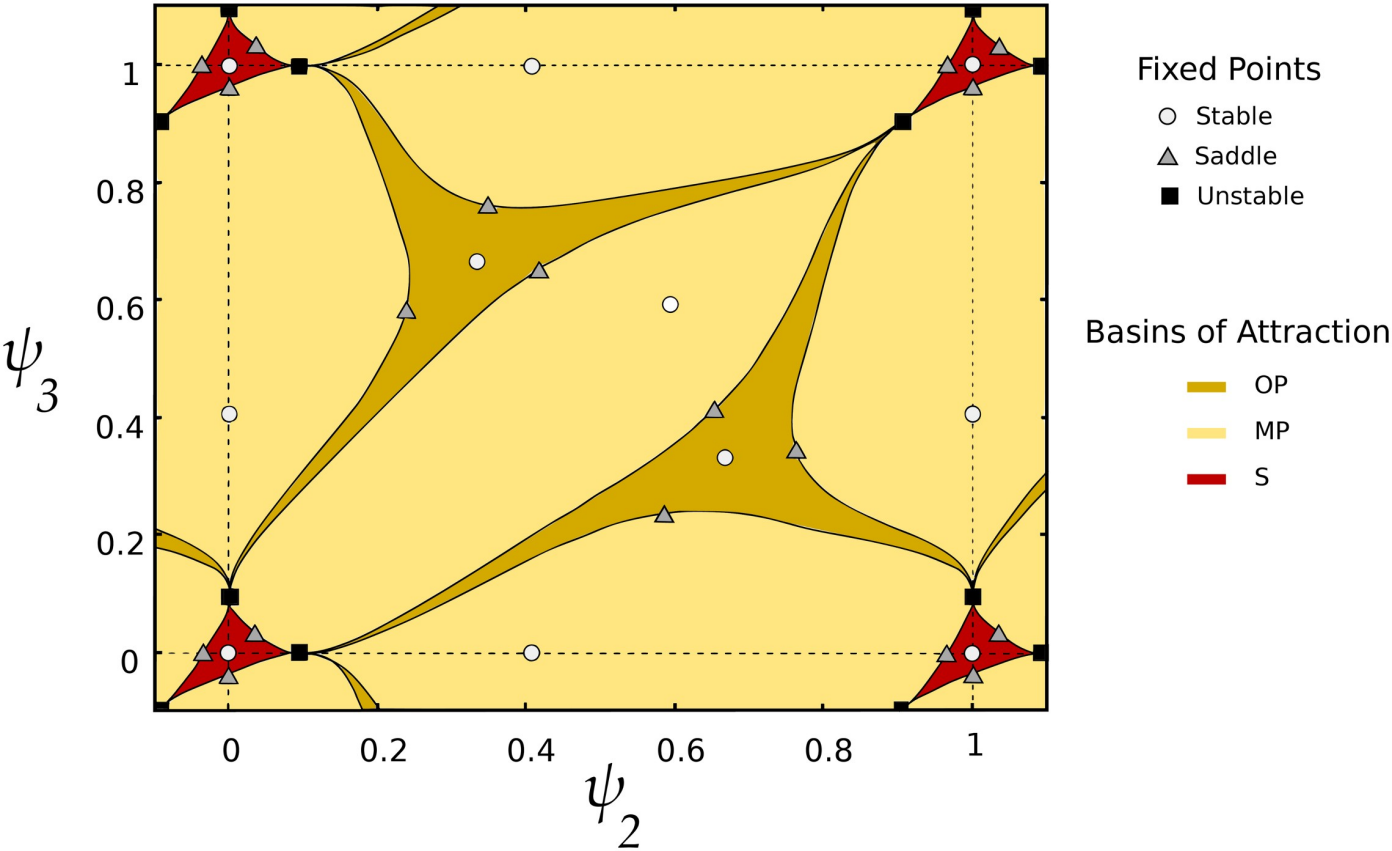
Basins of attraction for the case of weak EI coupling with 3 active populations. When three populations are active (note, *ψ*_*i*_ is the phase of population *i* relative to *θ*_1_), OP, S, and MP states all have open sets as basins of attraction, as we found in the full model. These basins are defined by the stable manifolds of saddle points, as shown.

With just EI or IE coupling, we can obtain all three relevant states of interest (OP, S, and MP). In Fig 7, we see the basins of attraction with only *c*_*ei*_ coupling (cf. Fig 2 in Horn and Opher 1996 [39]). Adding EE to EI coupling (*c*_*e*_ = 0.1*c*_*ei*_) predictably increases the basin of attraction of the synchronous state. Based on the geometry we see in Fig 7, we might also expect transitions between regions that share boundary curves to be easy to realize (e.g., the S and MP regions), and transitions between regions that only share isolated boundary points (the S and OP regions) to be more difficult to realize, if even possible. In fact, we found just such a correspondence when we more thoroughly examined accessible states (see *Accessible operations*).

### Biological considerations and applications

In *Mixed-phase oscillations: Synchronous and out-of-phase*, we showed that our model is able to support complex and stable oscillatory modes that may be relevant to different scenarios in working memory. Here, we explore further aspects of these oscillations that may be relevant to cognitive processes and empirical findings in working memory tasks. For example, in working memory, rapid transitions between activated states must occur in order to establish and adjust the relationships of items that are being held in memory. Thus, we describe how the patterns of synchronization that emerge from the model are rich enough to accommodate many aspects of the current understanding of both feature and variable binding from a working memory standpoint. We then examine in greater detail what transitions are in fact possible within the model. Finally, we describe the frequency relationships that naturally emerge in the model and how these compare to experimental findings of power spectra dynamics in large-scale recordings during working memory tasks.

#### Feature binding

**Fig 8:**
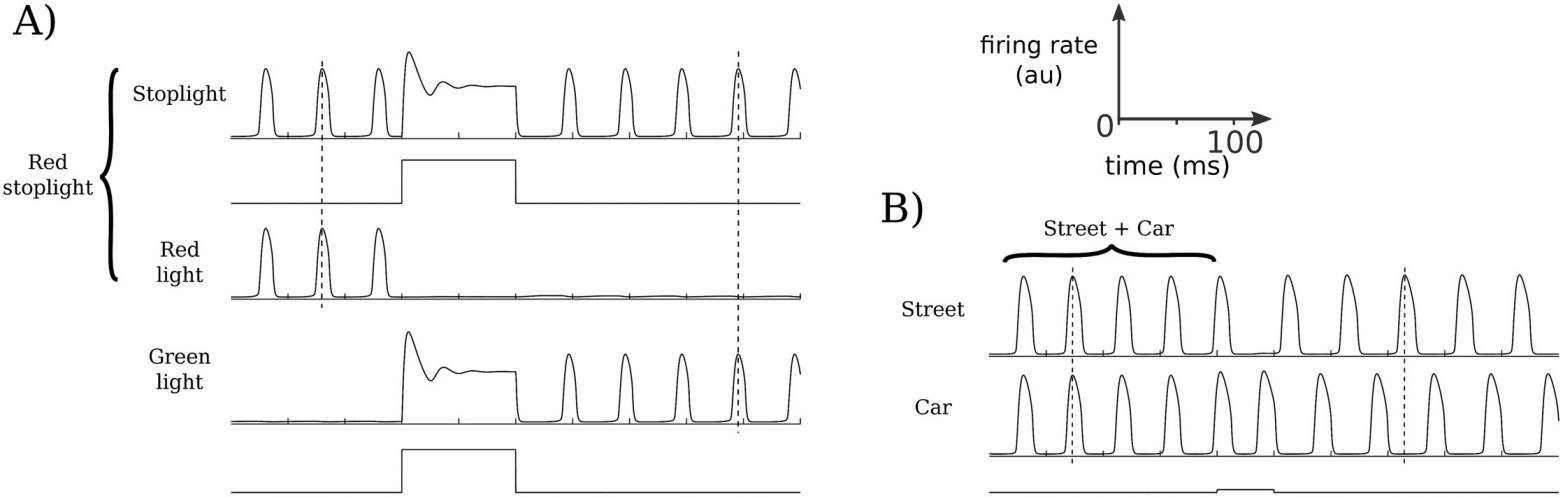
Feature binding examples. For simplicity, memoranda that may require several different bound features are represented as a single oscillating population (e.g., a street). (A) Feature binding can be achieved through the synchronous firing of the individual features as in the left half of (A). A new input causes an unbinding and a new binding of an object with a modified feature. For example, the initial binding might represent a *red stoplight*. The light changes to green, causing the *stoplight* to bind with *green light*. (B) Feature binding in which a new input (e.g., focus) results in the decoupling of a single memorandum (e.g., some background such as a *street* and stationary *car*). For example, the *car* is observed to begin moving, and so is now perceived as a separate object from the *street*.

Dynamic binding is required in working memory in order to quickly associate and dissociate different elements together [40], such as different features of an item in the environment. In the context of our model, this means that items must be able to be both synchronized and desynchronized in response to different stimuli. We have seen above in Fig 4 how synchronization may occur via either simultaneous (Fig 4A) or sequential (Fig 4B and 4C) selective stimuli. Such synchronization allows different features to bind together into one item in memory. In Fig 8, we see two situations in which the patterns of synchrony change as the associations between different features change. For example, if a red traffic light turns green (Fig 8A), *red light* can unbind from *stoplight* (and indeed may become quiescent, as in this example), while *green light* then binds to *stoplight* as the two associated neuronal populations synchronize. Alternatively, an observer may perceive the static environment in a unified manner, so that the car at the red light and the street, for example, are bound together (Fig 8B). Once the light turns green, the car begins to move, so that *car* and *street*, for example, unbind and are represented as distinct items in memory. Thus, attentional mechanisms or environmental changes may act to change the patterns of synchronization, and thus of the binding of different items in working memory, by stimulating either quiescent or already-active populations.

#### Variable binding

**Fig 9:**
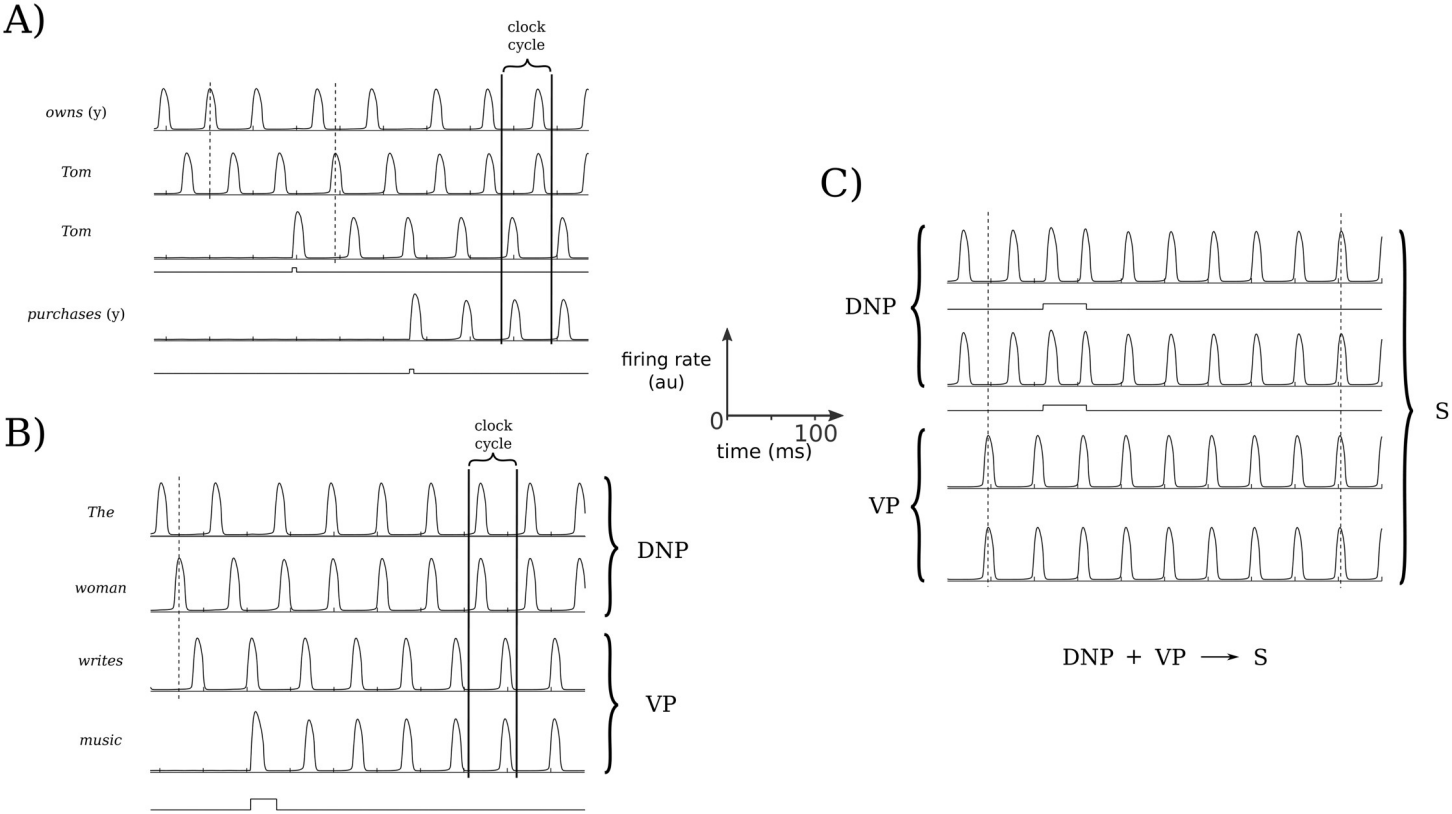
Variable binding, and sentence construction examples. Specific sequential inputs result in a cascade of bindings that emerge, following one another within a single clock cycle. (A) A fragment of the working memory system demonstrating how an inference emerges and is established in working memory. Here, the predicate calculus rule examined is purchases(*x*, *y*) ⇒ owns(*x*, *y*) (from an example in Feldman [12]). After a query is made (“Does Tom own *The Awakening*?”), a statement is provided, “Tom purchased *The Awakening*”. This statement first activates a second instantiation of *Tom*, and then the verb *purchases*(y), causing *owns*(y) and *Tom* to synchronize so that the inference is made. (B) An illustration of that same combinatorial structure is shown as it could apply towards a mechanistic realization of a phrase structure grammar. This could be based upon an already established or innate structure in the cortex. Upon reading the sentence, the words are sequentially input and the appropriate nodes are activated and bound in working memory, forming a determinate noun phrase (DNP) and a verb phrase (VP). For clarity, bindings between nodes and variables (i.e., words here) are not explicitly illustrated. (C) A final binding occurs, as the components of the DNP receive selective and equal simultaneous stimuli, binding all of the components together to form a sentence (S).

The combinatorial dynamics emerging within the model are rich enough to produce variable binding as is characteristic, for example, of language and abstract reasoning within the context of working memory. Below, we illustrate some simple examples from the model consistent with the most expected and widespread approaches to variable binding and language (e.g., the SHRUTI inference network [41]). These utilize temporal phase binding and represent simple predicate calculus rules. Consider the predicate calculus rule

purchases(x,y)⇒owns(x,y),

where *x* and *y* represent variables in the above rule, and a Boolean query (for example, as presented in Feldman [12]), such as “Does Tom (*x*) own a book (*y*)”. In most common inference network models, for example, SHRUTI [42], separate clock phases are assigned to the pairings of *Tom* with *owns* and *book* with *owns*. *Tom* here represents a variable agent who could come to own something (a *book*, for example) through *purchases*. There are other possibilities, of course, and they could all be linked or synchronized with the *owns* relation. We note that while a mediator circuit is implicated in the SHRUTI inference network, here the relevant association may occur in a single step as a result of synchronous firing due to temporal associations and the pattern of input, as shown in Fig 9. The active populations in working memory could represent an *owns* node (e.g., the active population in line 1 of Fig 9A), and *Tom* (as an agent node; e.g., the active populations in lines 2 and 3). The active populations of *Tom* and *book* then become associated and paired as a result of the activation of a node or population representing *purchases* in working memory (active population in line 4 of Fig 9A). Then, over several clock cycles “Tom owns a book” becomes established, or possibly instantly as here with the activation of the *purchases* node (i.e., population). Additional populations could become activated and linked via synchronization, such as “Tom purchases *The Awakening*” where *The Awakening* would become synchronized with a *book* node. We note that, whereas *Tom* requires two populations to be active in this example in order to form distinct bindings with both *purchases* and *owns*, different, *n*: *m* locking ratios or aperiodic oscillatory dynamics could allow for such unambiguous bindings while just one population is maintained active for *Tom*, as we touch on at the end of *Discussion*.

We can also consider how the combinatorics allowed through the present model’s dynamics may apply in language by illustrating how they may facilitate the presence of grammars. We note that the predicate-argument structure used above (e.g., owns(*x*, *y*)) fits well within some dependency grammars. However, the model is agnostic to any particular type of grammar, and we now consider an example as might be implemented within a phrase structure grammar, illustrated in Fig 9B and 9C. The words are represented by the activation of populations (presumably stored in the structure of networks in long-term memory) and become synchronized with the appropriate activated nodes. For clarity, we have simplified the example so that the nodes, and therefore the bindings of words to nodes, are not displayed. For example, a determiner node is first activated and then bound to *the*, but in Fig 9B we only show the activity of the population corresponding to *the*. A noun node is then activated, which is bound to the variable *woman*. A verb node is activated next, which is bound to *writes*. Finally a second noun node is activated and then bound to *music*. This results in the binding of the determiner node and noun node of the subject into a determinate noun phrase (displaying the phrase structure rule D + N → DNP), and within a single clock cycle the dynamic binding of the verb with the direct object to form a verb phrase (V + N → VP). These states are activated sequentially within a single clock cycle, and thus could be recognized as a grammatical sentence maintained in working memory based on the phrase structure rule that a DNP followed by a VP produces a grammatical sentence S. Alternatively, the sequential DNP and VP representations could be input with a subsequent final synchronization of the DNP and VP taking place to produce S, and recognized as a grammatical sentence that is maintained in working memory, as illustrated in Fig 9C.

Ungrammatical sentences could be recognized when bindings occur that do not correspond to valid production rules. We do not consider here the specific mechanism or details by which the particular variables become bound, but rather show how they can dynamically emerge within working memory representations. In principle this could arise via some mapping and closeness of the associations in that cortical map, “hardwired” architectural associations in long-term memory, or some combination.

#### Accessible operations

We have shown that the dynamics of our model map onto a number of working memory and binding examples. Indeed, it appears that the relevant attractor patterns are exactly the combinatorial possibilities, limited by the network size and the number of MP patterns available. Beyond ascertaining what states are available to the network, we are further interested in how the network can transition from one pattern to another. For example, in Fig 2B we see that if a third, inactive population is stimulated, the network can transition from 2 OP populations to 3 OP populations. We will consider this to be an operation available to the network; i.e., the operation of transitioning from 2 OP populations to 3 OP populations by providing a stimulus of the right strength and timing (see Methods for further protocol detail). Different patterns may require a different number of operations to attain from a given starting pattern. We refer to a transition that requires *n* selective, sequential stimuli to instantiate as an *n*th-order operation. We may assess the capabilities, and thus to some degree the plausibility, of the model more systematically by delimiting the available operations. Determining whether a pattern is accessible through a first-order or a higher-order operation may also allow for predictions to be made from the model, such as the timings involved in certain cognitive processes. We restrict the number of active populations to either two (diads) or three (triads). Our parameter choices are as above, so that WTA is always an accessible state. Thus, if we begin with a diad or triad, the only resultant patterns of interest are also diads and triads.

**Fig 10:**
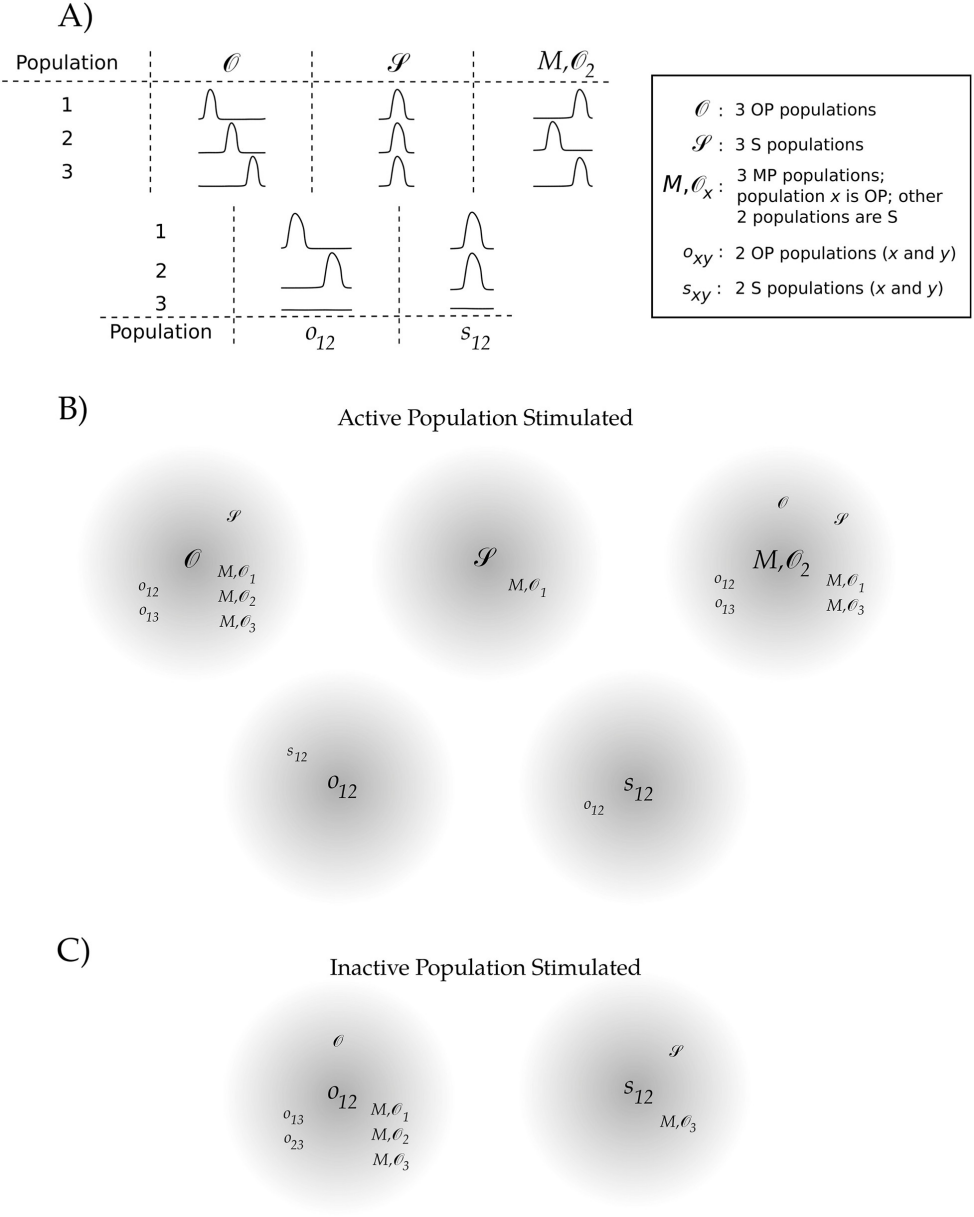
Accessible diad and triad operations. (A) Using *N* = 5, a stimulus is given to each of the activity patterns shown. For (B) and (C), the starting activity pattern is indicated in the center of the gray circles, while the subscripts indicate which population was stimulated. The observed resultant activity patterns are indicated in smaller text around the circumference of the gray circles. For (B), one of the already-active populations, population 1, was stimulated, whereas an inactive population, population 3, was stimulated for (C).

Fig 10 shows what operations related to diads and triads are available to the network, under the limitations described above and in *Methods*. In each gray circle, 1st-order operations are displayed; we may determine available higher-order operations by stringing together sequences of 1st-order operations. For example, we may transition from an OP diad to an S triad as a 2nd-order operation by stimulating an inactive population so that the network first transitions to an OP triad, and then stimulating an active population so that the network then transitions to an S triad. In particular, examining Fig 10B and 10C reveals that the network may transition from any diad or triad to any other (through transitions involving only other diads and triads) using no higher than a 3rd-order operation (i.e., no more than 3 selective, sequential excitatory stimuli are required).

Since the stimulus is excitatory, the stimulated population will remain active. Thus, every beginning pattern will have at least two resultant patterns that will not be accessible for a given selected population that receives a stimulus (e.g., beginning with *O* and stimulating population 1 will ensure that *s*_23_ and *o*_23_ are not accessible—see Fig 10A for a notation key). By changing the population that receives the stimulus, other patterns become available in a way that is obvious by inspecting Fig 10. For example, we see in Fig 10B that an MP triad and OP diads and triads can transition to most of the remaining diads and triads: Discounting order, there are 5 possible triads and 6 possible diads; disregarding which population receives the stimulus, we see that both an MP and an OP triad can directly transition to 6 of the 10 patterns that are different from themselves. By contrast, the S populations have fewer populations accessible by 1st-order operations. This is in part an outcome of maintaining a uniform connectivity. In order to differentially affect two S populations, one of them must receive a stimulus, as any other population will affect the two S populations symmetrically. So, for example, it is as expected that the only accessible patterns for 3 S populations are the ones consisting of 3 MP populations where the selectively stimulated population oscillates out-of-phase with the remaining two. The results for *s*_12_ as shown are likewise entirely expected.

One of the patterns that is difficult to directly obtain from a diad or triad is an S diad (e.g., *s*_12_). We were only able to obtain this pattern by stimulating one of the active populations in an OP diad (e.g., *o*_12_). Thus, going from any other pattern to an S diad requires at least a second order operation. For example, transitioning from an S triad to an S diad is at least a third order operation: With the first stimulus the network may transition to an MP triad, with the second the network may transition to an OP diad, and with the third the network may finally transition to an S diad. We note that there may be multiple paths that allow one pattern to evolve to another pattern. For example, an alternative route from the S triad to diad would be through a WTA scenario: A strong first stimulus can cause the selected population to quench the activity of the other populations, and a second stimulus to a nonactive population could cause the network to transition to an S diad.

These results show that there are a number of options to get from one pattern to another for two and three active populations, even with strong limitations on the network architecture and the stimulus protocol. For the operations that take triads to triads (as shown in Fig 10B, top), we note that the accessible operations are almost exactly what we would have predicted from the weak coupling analysis. That is, as we mention in *Weak coupling*, we expect transitions between basins of attraction with boundaries given as curves in Fig 7 (i.e., between OP and MP basins and between MP and S basins) to be easier to realize, and transitions between basins of attraction with boundaries given as points (as in the unstable fixed points separating OP and S basins) to be more difficult to realize. Indeed, we found it easy to transition between OP and MP states and between S and MP states, and found it more difficult to transition between OP and S states (note that we were unable to transition from S to O within the constraints given in *Methods*, while there is only a very narrow 7ms window that allows us to transition from O to S, just within our stated protocol). It may be of interest in future work to loosen some of the above restrictions (for example, examining heterogeneous networks or having the stimulus also drive the inhibitory components), and to further quantify the level of difficulty for a particular transition (e.g., some transitions are much less dependent on the stimulus parameters than others).

#### Frequencies

**Fig 11:**
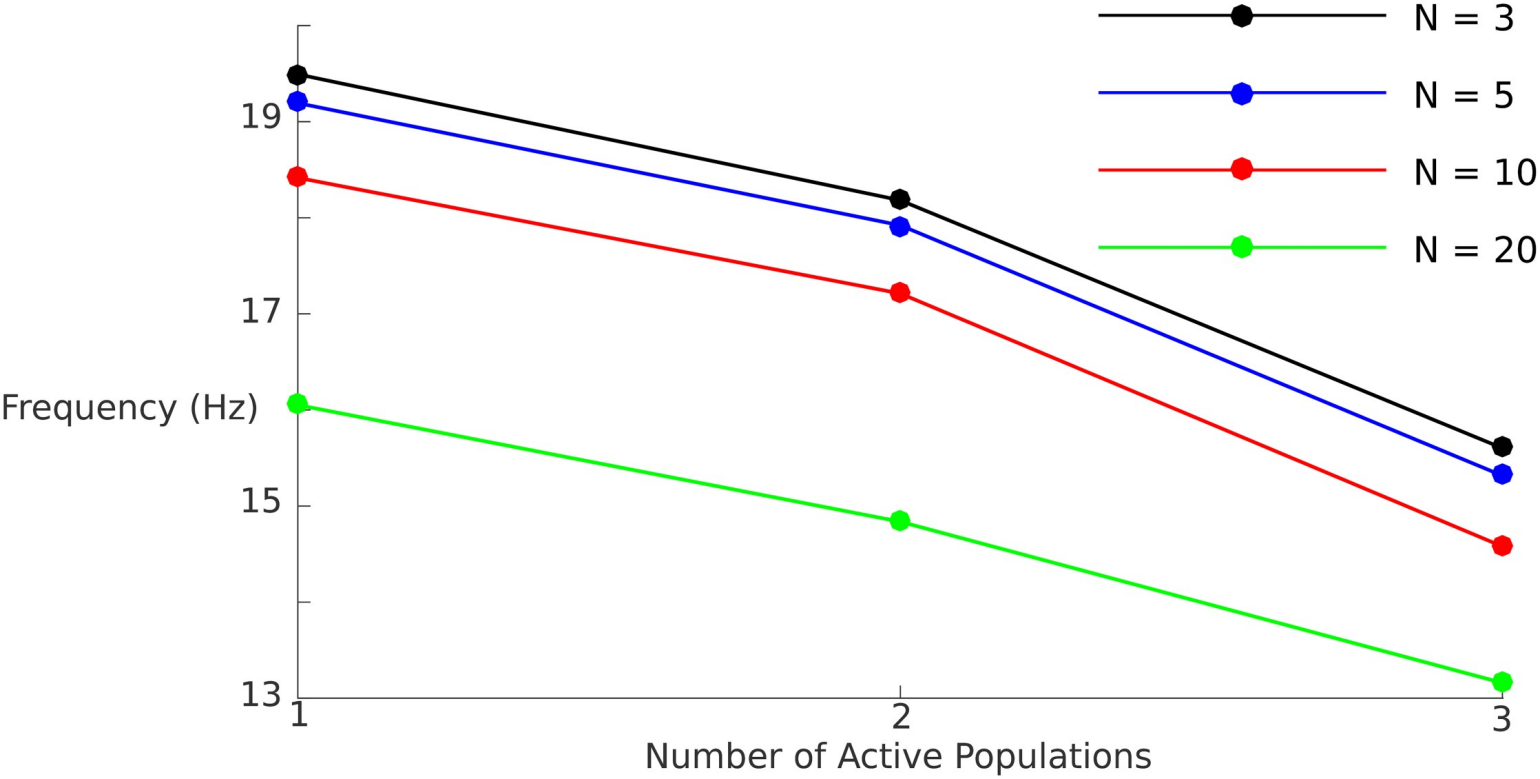
Frequency of population oscillation for different network sizes *N*. We examine the effect of increasing both system size and number of active OP populations on the oscillation frequency. In both cases, the frequency decreases monotonically. The frequency of interest may also be that based on the time between peaks of active populations (e.g., the time between the peak firing rates of population 1 and population 2 in a given network). This “interpopulation frequency” may be obtained by multiplying the given population by the number of active populations. For example, the interpopulation frequency for a network size of 20 when 2 populations are active would be approximately 30 Hz.

In constructing our model network, we chose parameters that were biologically plausible and further found constraints on these parameters that allowed OP, S, and MP modes and transitions between them that are relevant to working memory situations. While we did not tune parameters in order to fit certain frequency bands that have been found to be of potential relevance to working memory and binding, we indeed observed some correspondence between these and the frequency relationships in the model network. For example, if we have populations that oscillate OP, then as each population becomes active, the oscillation frequency of the network as a whole (or, equivalently, of each individual population) decreases, as illustrated in Fig 11. If the number of active OP populations remains constant, the frequency of the network oscillations also decreases with increasing network size. Thus, for example, if there are three active populations with OP dynamics, each population oscillates at just over 15 Hz for a network with *N* = 3, and at approximately 13 Hz for a network with *N* = 20. We see in Fig 11 that if either *N* or the number of active populations corresponding to distinct memoranda increases, the frequency of oscillations for any individual population trends downward towards the alpha band.

Since we are considering OP dynamics, we also note that the period between successive peaks in the case when, for example, three populations are active, is one third the overall period of the network. We may refer to the associated measure as the “interpopulation period/frequency”. As an example, let us consider a network size of *N* = 20 with three active OP populations. As mentioned above, the network oscillation, and thus the oscillation of any particular population, is 13 Hz. However, since three populations are active OP, the interpopulation frequency is 39 Hz. Depending on the distances between these active populations of neurons and the spatial resolution of the measurement in an experimental setting, then, increased activity near the alpha and/or gamma band may be detected. Greater numbers of populations that oscillate S may cause increased power near the alpha spectrum, since the interpopulation frequency would not increase. Increases in power in gamma frequencies and in frequencies near alpha are both consistent with neurophysiological experiments that have reported increases in gamma or alpha contributions with working memory tasks and increasing working memory load [6, 23–25, 43–47]. In addition, increased alpha-band oscillations have been reported to play an important role in mental processes related to attention and memory [48–53].

## Discussion

We presented an oscillatory firing rate network model that can represent both distinct and bound items in working memory. The oscillations provide a mechanism to separate or bind these items by utilizing the phase information of the oscillations, resulting in groupings of synchronous and asynchronous populations that represent multi-item, multi-feature memories. By framing our mean-field model within a dynamical systems context, we were able to study how the oscillatory solutions arise as functions of key network parameters, such as the coupling strengths and synaptic timescales. We were also able to rapidly simulate various scenarios of interest, which allowed us to explore different oscillatory states and transitions between them that may be relevant to working memory and binding.

Beginning with a spiking network, we derived a simple mean-field model. In addition to the fast AMPA and slow GABA synapses that are normally modeled, we included slower excitatory NMDA synapses, which have been implicated in the persistent activation of neurons during information retention in working memory tasks. The NMDA synapses allow for bistability in our model, so that only transient stimuli, applied to the excitatory components, are required to trigger the active oscillatory states, consistent with experimental findings and other modeling efforts that have employed NMDA. While others have examined oscillatory models in working memory and binding [36, 38, 39, 54–66], to our knowledge none have employed NMDA as a mechanism for bistability, used numerical continuation to define regions of parameter space that allow for the oscillations of interest, or explored in detail the combinatorially rich oscillatory states and transitions between them.

We found attracting states involving synchronous (S) and out-of-phase (OP) oscillations, as well mixed-phase (MP) states where some populations oscillated S and others OP. Numerical continuation defined regions in parameter space where they exist stably. The mutual inhibition between populations (*c*_*ei*_) both facilitated OP states through competition and also limited the number of populations that could oscillate. We found a wide range of biologically plausible parameter values that allow three populations to oscillate mutually OP, in line with the 3–5 item working memory capacity found across different modalities [7, 12, 67]. While *c*_*ei*_ strongly affected the number of populations that could oscillate OP, it had only a weak effect on the number of populations that could oscillate S. The model provides a natural explanation for the capacity of working memory, apart from the system’s bifurcations discussed above in *Out-of-phase oscillations and distinct memoranda*, in that the excitatory coupling *c*_*e*_ can greatly increase the basin of attraction of synchronous states, as we show explicitly in the case of weak coupling. Thus, two populations may only get so close before they will tend towards synchrony, implying there may only be a finite number of OP populations. However, for *c*_*e*_ too large, the network is only attracted to a bulk oscillatory state. While synchrony is desirable in some binding situations, too much synchrony can be pathological, so it is necessary to maintain low-level excitatory connections between distinct populations.

We explored a number of different scenarios by adjusting the stimuli parameters. We found by stimulating either inactive or active populations that the network could rapidly transition between accessible oscillatory states. Thus, depending on the strength and timing of the stimuli, populations could quickly synchronize, desynchronize, or become quiescent. All of these operations are necessary for binding and cognitive processes within working memory. We found many different sets of dynamics that are relevant to binding, and the flexibility of the network lends itself to linguistic demands. Thus, our network may provide a framework to realize certain ideas in grammars, sentence construction, and simple predicate calculus. In particular, the attracting S, OP, and MP states and the response speeds to selective stimuli allow our network to provide a versatile underlying skeleton to realize such binding scenarios as outlined in a connectionist setting in, for example, Shastri et al. [41]

More generally, the oscillatory activity provides basic properties by which executive processes can direct ongoing cognitive operations through the control of the contents of declarative or procedural working memory. The dynamics meet the potentially opposing demands of this control by providing a mechanism for quickly establishing and maintaining new structures or representations by both forming strong bindings that are resistant to interference and retaining the ability to rapidly dissolve those bindings in order to update the contents of working memory, or remove content from working memory that is no longer relevant. Thus, a primary result in this work is that binding via synchronization between populations that represent working memory elements, the number of arrangements of which appears limited only by the number of permutations of active populations, may be rapidly and stably formed as well as dissolved to form new structures from other populations that represent elements in long-term memory. However, while all arrangements of the active populations appear possible, different numbers of operations (e.g., activations or deactivations) are necessary to reach particular states from each other. This could be a factor involved in different lag times in the performance of different types of working memory tasks, as observed in numerous studies [17, 68–71].

We note that in our model, working memory and binding depend on the onset time or phase presentation of “stimuli”. However, these stimuli do not necessarily represent direct stimuli, but rather could be inferred to be filtered through some control circuitry not explicitly addressed in the present model. Nonetheless, our model does require precise phase timing control of selective stimuli that can affect whether items are perceived as distinct or bound. The existence of such precise timing control is essentially a prediction of the model. However, there is some evidence that indicates such finely tuned temporal control is plausible. As we mentioned in *Methods*, some pyramidal neurons and interneurons lock to two different phases of a gamma oscillation, allowing as little as 6–7ms temporal resolution [34], and synchronization between neurons has been shown to occur within a time window of around 10ms [35]. Even for direct external inputs in working memory and binding, it has been shown that sequential inputs of stimuli, in conjunction with other stimulus characteristics (e.g., spatial characteristics), lead to increased misbinding if presented with a sufficiently short duration. While the functioning of visual working memory has been shown to be independent of the cortical spacing between memoranda [72, 73], studies of multidimensional perceptual interaction have shown that presenting stimuli in short time windows results in different perceptions and bindings. For example, the pitch and loudness of a 200ms tone is experienced differently from a 50ms tone of the same pitch or loudness [74, 75]. Furthermore, a significant amount of work has shown that binding and differentiation of distinct items in memory depend in part on timings associated with the gamma band [23, 25, 27, 35, 37, 76]. These considerations make the existence of such short phase precision plausible.

Oscillatory models have been developed both in the context of working memory [36, 54–56] and of binding [38, 57–59], and models—often with an eye towards image processing—have employed the distinction between bound and distinct objects as synchronous or asynchronous oscillations [38, 59–62, 66]. These models tend to be spiking networks, appeal to cross-frequency coupling (e.g., theta-gamma codings), provide unrealistic connections (e.g., delayed self-inhibition for excitatory elements), use delays or constant inputs to produce persistent oscillatory activity, or employ structured architectures (e.g., using Hopfield networks, Hebbian rules, or pre-wired assemblies).

The line of work that is the closest to the present study also utilized Wilson-Cowan-type networks [39, 55, 63–65]. By incorporating common inhibition, dynamic thresholds, and sustained inputs, they could obtain asynchronous oscillations. The dynamic thresholds (which could instead be interpreted as linear inhibitory neurons) induced oscillatory activity, global (nonlinear) inhibition allowed for competitive dynamics that led to asynchronous oscillations, and continuous inputs kept the selected populations active (or quiescent at or near a low fixed point for negative-valued inputs). In Horn and Usher [64] and Horn and Opher [65] in particular, correlated noise was added to the inputs to obtain synchronous oscillations (uncorrelated noise resulted in asynchronous oscillations). In Horn and Opher [39], an explanation that may be relevant to the network we consider was provided for the limit to the number of active asynchronous populations in terms of the subharmonic solutions that could be obtained by driving a single oscillator at greater frequencies. However, the mechanisms our model employs for oscillatory dynamics, sustained activity, and competitive asynchronous and synchronous oscillations are all distinct from this interesting series of papers.

Indeed, our model differs in important aspects from all of the above-cited body of work. We draw on evidence that implicates NMDA in the persistent activity in neurons associated with working memory, incorporating simplified NMDA synapses to produce persistent activation [2, 28–31]. Thus, transient inputs result in sustained activity, in line with neurophysiological studies. Our network has strong local and weaker global inhibition and excitation with simple uniform all-to-all connectivity that, importantly, allows any of the populations to be in- or out-of-phase with each other. Finally, we consider the ensemble activity through a mean-field model that we develop from a spiking quadratic integrate-and-fire network. On the one hand, this allows us to relate the mean-field dynamics to those of the underlying spiking network. On the other hand, the mean-field model has the advantage of being posed in a more mathematically tractable form than spiking models, allowing us to analyze the dependence of states on different network parameters through numerical continuation methods. The reduced computational requirements of the mean-field network also free us to explore more of the model’s rich dynamical behaviors, and we have included several examples of interesting and relevant working memory and binding activities.

Increased memory loads during working memory tasks have been associated with increases in the spectral power in both gamma and the lower (e.g., alpha) frequency bands in, for example, EEG and MEG recordings [6, 23–25, 43–47]. Neuronal networks may select for (coherent) oscillatory signals in the gamma range in particular [77]. In our model, the frequency of the oscillations depends both on system parameters and network-level interactions. For most parameter values, the peak-to-peak period of oscillations within a given working memory cycle corresponds to either gamma or beta band oscillations. As more populations become active OP, however, while the overall peak-to-peak frequency remains in the higher frequency bands (gamma or high beta) in a working memory cycle, the peak-to-peak frequency of each separately active population firing within the cycle decreases towards lower bands. As more populations become active S, they may increase the contribution of either lower or higher frequencies. Thus, depending on the spatial distribution of the populations active in the working memory cycle, either gamma power may increase or else a “downshift” in measured frequencies (e.g., an increase in activity in low beta or near the alpha band) may be expected with increasing working memory load.

We focused on the simplest cases of uniform all-to-all coupling and periodic oscillators, allowing for clear network state classification. It would be interesting to explore more realistic heterogeneous coupling, including distance-based. Preliminary results have shown interesting phase relationships can develop, such as 2:1 frequency locking. Placing the oscillators into parameter regimes that allow for quasiperiodic or chaotic regimes (as seen to exist in E-I Wilson-Cowan networks in [78]) may also allow for even richer phase relationships as in the spiking networks in Raffone and van Leeuwen [59]. For example, a population may be in some sense partially synchronous with several other populations so that a feature (such as a color) could be associated with several distinct objects that themselves oscillate asynchronously.

## Supporting information

S1 Text Mean field model motivation and spiking network comparison. (PDF)

S2 Text The *u*, *v*, *n* system. (PDF)

S3 Text Weak coupling analysis. (PDF)

S4 Text Change in OP and S dynamics with varying coupling strengths and synaptic timescales. (PDF)

## References

[1] Fuster JM, Alexander GE. Neuron activity related to short-term memory. Science. 1971; 173(3997):652–654. 10.1126/science.173.3997.652 4998337 10.1126/science.173.3997.652

[2] Wang XJ. Synaptic basis of cortical persistent activity: The importance of NMDA receptors to working memory. Journal of Neuroscience. 1999; 19(21):9587–9603. 10.1523/JNEUROSCI.19-21-09587.1999 10531461 10.1523/JNEUROSCI.19-21-09587.1999 PMC6782911

[3] Fuster JM, Bodner M, Kroger JK. Cross-modal and cross-temporal association in neurons of frontal cortex. Nature. 2000; 405(6784):347 10.1038/35012613 10830963 10.1038/35012613

[4] Pesaran B, Pezaris JS, Sahani M, Mitra PP, Andersen RA. Temporal structure in neuronal activity during working memory in macaque parietal cortex. Nature Neuroscience. 2002; 5(8):805–812. 10.1038/nn890 12134152 10.1038/nn890

[5] Shafi M, Zhou Y, Quintana J, Chow C, Fuster J, Bodner M. Variability in neuronal activity in primate cortex during working memory tasks. Neuroscience. 2007; 146(3):1082–1108. 10.1016/j.neuroscience.2006.12.072 17418956 10.1016/j.neuroscience.2006.12.072

[6] Ku Y, Bodner M, Zhou YD. Prefrontal cortex and sensory cortices during working memory: Quantity and quality. Neuroscience Bulletin. 2015; 31(2):175–182. 10.1007/s12264-014-1503-7 25732526 10.1007/s12264-014-1503-7 PMC5563698

[7] Cowan N. The magical number 4 in short-term memory: A reconsideration of mental storage capacity. Behavioral and Brain Sciences. 2001; 24(1):87–114. 10.1017/S0140525X01003922 11515286 10.1017/s0140525x01003922

[8] Baddeley A. Working memory: Looking back and looking forward. Nature Reviews Neuroscience. 2003; 4(10):829 10.1038/nrn1201 14523382 10.1038/nrn1201

[9] Luck SJ, Vogel EK. The capacity of visual working memory for features and conjunctions. Nature. 1997; 390(6657):279. 9384378 10.1038/36846

[10] Vogel EK, Woodman GF, Luck SJ. Storage of features, conjunctions, and objects in visual working memory. Journal of Experimental Psychology: Human Perception and Performance. 2001; 27(1):92 11248943 10.1037//0096-1523.27.1.92

[11] Todd JJ, Marois R. Capacity limit of visual short-term memory in human posterior parietal cortex. Nature. 2004; 428(6984):751 10.1038/nature02466 15085133 10.1038/nature02466

[12] Feldman J. The neural binding problem(s). Cognitive Neurodynamics. 2013; 7(1):1–11. 10.1007/s11571-012-9219-8 24427186 10.1007/s11571-012-9219-8 PMC3538094

[13] von der Malsburg C. The correlation theory of brain function In: Models of Neural Networks. Springer; 1994 p. 95–119.

[14] Singer W, Gray CM. Visual feature integration and the temporal correlation hypothesis. Annual Review of Neuroscience. 1995; 18(1):555–586. 10.1146/annurev.ne.18.030195.003011 7605074 10.1146/annurev.ne.18.030195.003011

[15] Singer W. Neuronal synchrony: A versatile code for the definition of relations? Neuron. 1999; 24(1):49–65. 10.1016/S0896-6273(00)80821-1 10677026 10.1016/s0896-6273(00)80821-1

[16] Engel AK, König P, Singer W. Direct physiological evidence for scene segmentation by temporal coding. Proceedings of the National Academy of Sciences. 1991; 88(20):9136–9140. 10.1073/pnas.88.20.9136 10.1073/pnas.88.20.9136 PMC52667 1924376

[17] Kessler Y, Meiran N. All updateable objects in working memory are updated whenever any of them are modified: Evidence from the memory updating paradigm. Journal of Experimental Psychology: Learning, Memory, and Cognition. 2006; 32(3):570 10.1037/0278-7393.32.3.570 16719667 10.1037/0278-7393.32.3.570

[18] Fries P, Reynolds JH, Rorie AE, Desimone R. Modulation of oscillatory neuronal synchronization by selective visual attention. Science. 2001; 291(5508):1560–1563. 11222864 10.1126/science.1055465

[19] Cowan N, Blume CL, Saults JS. Attention to attributes and objects in working memory. Journal of Experimental Psychology: Learning, Memory, and Cognition. 2013; 39(3):731 10.1037/a0029687 22905929 10.1037/a0029687 PMC3825193

[20] Hardman KO, Cowan N. Remembering complex objects in visual working memory: Do capacity limits restrict objects or features? Journal of Experimental Psychology: Learning, Memory, and Cognition. 2015; 41(2):325 10.1037/xlm0000031 25089739 10.1037/xlm0000031 PMC4317397

[21] Klimesch W. EEG alpha and theta oscillations reflect cognitive and memory performance: A review and analysis. Brain Research Reviews. 1999; 29(2-3):169–195. 10.1016/S0165-0173(98)00056-3 10209231 10.1016/s0165-0173(98)00056-3

[22] Başar E, Başar-Eroglu C, Karakaş S, Schürmann M. Gamma, alpha, delta, and theta oscillations govern cognitive processes. International Journal of Psychophysiology. 2001; 39(2-3):241–248. 10.1016/S0167-8760(00)00145-8 11163901 10.1016/s0167-8760(00)00145-8

[23] Howard MW, Rizzuto DS, Caplan JB, Madsen JR, Lisman J, Aschenbrenner-Scheibe R, Schulze-Bonhage A, Kahana MJ Gamma oscillations correlate with working memory load in humans. Cerebral Cortex. 2003; 13(12):1369–1374. 10.1093/cercor/bhg084 14615302 10.1093/cercor/bhg084

[24] Jensen O, Gelfand J, Kounios J, Lisman JE. Oscillations in the alpha band (9–12 Hz) increase with memory load during retention in a short-term memory task. Cerebral Cortex. 2002; 12(8):877–882. 10.1093/cercor/12.8.877 12122036 10.1093/cercor/12.8.877

[25] Roux F, Uhlhaas PJ. Working memory and neural oscillations: Alpha–gamma versus theta–gamma codes for distinct WM information? Trends in Cognitive Sciences. 2014; 18(1):16–25. 10.1016/j.tics.2013.10.010 24268290 10.1016/j.tics.2013.10.010

[26] Verduzco-Flores S, Ermentrout B, Bodner M. From working memory to epilepsy: Dynamics of facilitation and inhibition in a cortical network. Chaos: An Interdisciplinary Journal of Nonlinear Science. 2009; 19(1):015115 10.1063/1.3080663 10.1063/1.3080663 19335019

[27] Lisman J. Working memory: The importance of theta and gamma oscillations. Current Biology. 2010; 20(11):R490–R492. 10.1016/j.cub.2010.04.011 20541499 10.1016/j.cub.2010.04.011

[28] Lisman JE, Fellous JM, Wang XJ. A role for NMDA-receptor channels in working memory. Nature Neuroscience. 1998; 1(4):273–275. 10.1038/1086 10195158 10.1038/1086

[29] Adler CM, Goldberg TE, Malhotra AK, Pickar D, Breier A. Effects of ketamine on thought disorder, working memory, and semantic memory in healthy volunteers. Biological Psychiatry. 1998; 43(11):811–816. 10.1016/S0006-3223(97)00556-8 9611670 10.1016/s0006-3223(97)00556-8

[30] Krystal JH, Abi-Saab W, Perry E, D’Souza DC, Liu N, Gueorguieva R, McDougall L, Hunsberger T, Belger A, Levine L, Breier A Preliminary evidence of attenuation of the disruptive effects of the NMDA glutamate receptor antagonist, ketamine, on working memory by pretreatment with the group II metabotropic glutamate receptor agonist, LY354740, in healthy human subjects. Psychopharmacology. 2005; 179(1):303–309. 10.1007/s00213-004-1982-8 15309376 10.1007/s00213-004-1982-8

[31] Compte A, Brunel N, Goldman-Rakic PS, Wang XJ. Synaptic mechanisms and network dynamics underlying spatial working memory in a cortical network model. Cerebral Cortex. 2000; 10(9):910–923. 10.1093/cercor/10.9.910 10982751 10.1093/cercor/10.9.910

[32] Ermentrout GB, Terman DH. Mathematical Foundations of Neuroscience. Vol. 35 Springer Science & Business Media; 2010.

[33] Ermentrout B. Simulating, Analyzing, and Animating Dynamical Systems: A Guide to XPPAUT for Researchers and Students. Vol. 14 SIAM; 2002.

[34] Senior TJ, Huxter JR, Allen K, O’Neill J, Csicsvari J. Gamma oscillatory firing reveals distinct populations of pyramidal cells in the CA1 region of the hippocampus. Journal of Neuroscience. 2008; 28(9):2274–2286. 10.1523/JNEUROSCI.4669-07.2008 18305260 10.1523/JNEUROSCI.4669-07.2008 PMC6671861

[35] Engel AK, Singer W. Temporal binding and the neural correlates of sensory awareness. Trends in Cognitive Sciences. 2001; 5(1):16–25. 10.1016/S1364-6613(00)01568-0 11164732 10.1016/s1364-6613(00)01568-0

[36] Lisman JE, Idiart MA. Storage of 7+/-2 short-term memories in oscillatory subcycles. Science. 1995; 267(5203):1512–1515. 10.1126/science.7878473 7878473 10.1126/science.7878473

[37] Lisman JE, Jensen O. The theta-gamma neural code. Neuron. 2013; 77(6):1002–1016. 10.1016/j.neuron.2013.03.007 23522038 10.1016/j.neuron.2013.03.007 PMC3648857

[38] Raffone A, Wolters G. A cortical mechanism for binding in visual working memory. Journal of Cognitive Neuroscience. 2001; 13(6):766–785. 10.1162/08989290152541430 11564321 10.1162/08989290152541430

[39] Horn D, Opher I. Temporal segmentation in a neural dynamic system. Neural Computation. 1996; 8(2):373–389. 10.1162/neco.1996.8.2.373 8581886 10.1162/neco.1996.8.2.373

[40] Oberauer K. Interference between storage and processing in working memory: Feature overwriting, not similarity-based competition. Memory & Cognition. 2009; 37(3):346–357. 10.3758/MC.37.3.346 19246349 10.3758/MC.37.3.346

[41] Shastri L, Ajjanagadde V. From simple associations to systematic reasoning: A connectionist representation of rules, variables and dynamic bindings using temporal synchrony. Behavioral and Brain Sciences. 1993; 16(3):417–451. 10.1017/S0140525X00030910

[42] Wendelken C, Shastri L. Multiple instantiation and rule mediation in SHRUTI. Connection Science. 2004; 16(3):211–217. 10.1080/09540090412331311932

[43] Tallon-Baudry C, Bertrand O, Peronnet F, Pernier J. Induced *γ*-band activity during the delay of a visual short-term memory task in humans. Journal of Neuroscience. 1998; 18(11):4244–4254. 10.1523/JNEUROSCI.18-11-04244.1998 9592102 10.1523/JNEUROSCI.18-11-04244.1998 PMC6792803

[44] Tallon C, Bertrand O, Bouchet P, Pernier J. Gamma-range activity evoked by coherent visual stimuli in humans. European Journal of Neuroscience. 1995; 7(6):1285–1291. 10.1111/j.1460-9568.1995.tb01118.x 7582101 10.1111/j.1460-9568.1995.tb01118.x

[45] Roux F, Wibral M, Mohr HM, Singer W, Uhlhaas PJ. Gamma-band activity in human prefrontal cortex codes for the number of relevant items maintained in working memory. Journal of Neuroscience. 2012; 32(36):12411–12420. 10.1523/JNEUROSCI.0421-12.2012 22956832 10.1523/JNEUROSCI.0421-12.2012 PMC6621256

[46] Kaiser J, Rahm B, Lutzenberger W. Temporal dynamics of stimulus-specific gamma-band activity components during auditory short-term memory. Neuroimage. 2009; 44(1):257–264. 10.1016/j.neuroimage.2008.08.018 18790066 10.1016/j.neuroimage.2008.08.018

[47] Lutzenberger W, Ripper B, Busse L, Birbaumer N, Kaiser J. Dynamics of gamma-band activity during an audiospatial working memory task in humans. Journal of Neuroscience. 2002; 22(13):5630–5638. 12097514 10.1523/JNEUROSCI.22-13-05630.2002 PMC6758237

[48] Jensen O, Bonnefond M, VanRullen R. An oscillatory mechanism for prioritizing salient unattended stimuli. Trends in Cognitive Sciences. 2012; 16(4):200–206. 10.1016/j.tics.2012.03.002 22436764 10.1016/j.tics.2012.03.002

[49] Voytek B, Canolty RT, Shestyuk A, Crone NE, Parvizi J, Knight RT. Shifts in gamma phase–amplitude coupling frequency from theta to alpha over posterior cortex during visual tasks. Frontiers in Human Neuroscience. 2010; 4 10.3389/fnhum.2010.00191 21060716 10.3389/fnhum.2010.00191 PMC2972699

[50] Medendorp WP, Kramer GF, Jensen O, Oostenveld R, Schoffelen JM, Fries P. Oscillatory activity in human parietal and occipital cortex shows hemispheric lateralization and memory effects in a delayed double-step saccade task. Cerebral Cortex. 2006; 17(10):2364–2374. 10.1093/cercor/bhl145 17190968 10.1093/cercor/bhl145

[51] Worden MS, Foxe JJ, Wang N, Simpson GV. Anticipatory biasing of visuospatial attention indexed by retinotopically specific-band electroencephalography increases over occipital cortex. Journal of Neuroscience. 2000; 20(RC63):1–6. 10704517 10.1523/JNEUROSCI.20-06-j0002.2000 PMC6772495

[52] Klimesch W, Sauseng P, Hanslmayr S. EEG alpha oscillations: The inhibition–timing hypothesis. Brain Research Reviews. 2007; 53(1):63–88. 10.1016/j.brainresrev.2006.06.003 16887192 10.1016/j.brainresrev.2006.06.003

[53] Klimesch W. Alpha-band oscillations, attention, and controlled access to stored information. Trends in Cognitive Sciences. 2012; 16(12):606–617. 10.1016/j.tics.2012.10.007 23141428 10.1016/j.tics.2012.10.007 PMC3507158

[54] Wang D, Buhmann J, von der Malsburg C. Pattern segmentation in associative memory. Neural Computation. 1990; 2(1):94–106. 10.1162/neco.1990.2.1.94

[55] Horn D, Usher M. Parallel activation of memories in an oscillatory neural network. Neural Computation. 1991; 3(1):31–43. 10.1162/neco.1991.3.1.31 10.1162/neco.1991.3.1.31 31141868

[56] Winder RK, Reggia JA, Weems SA, Bunting MF. An oscillatory Hebbian network model of short-term memory. Neural Computation. 2009; 21(3):741–761. 10.1162/neco.2008.02-08-715 18928370 10.1162/neco.2008.02-08-715

[57] König P, Schillen TB. Stimulus-dependent assembly formation of oscillatory responses: I. Synchronization. Neural Computation. 1991; 3(2):155–166. 10.1162/neco.1991.3.2.155 10.1162/neco.1991.3.2.155 31167303

[58] Sompolinsky H, Golomb D, Kleinfeld D. Global processing of visual stimuli in a neural network of coupled oscillators. Proceedings of the National Academy of Sciences. 1990; 87(18):7200–7204. 10.1073/pnas.87.18.7200 10.1073/pnas.87.18.7200 PMC54711 2402502

[59] Raffone A, van Leeuwen C. Dynamic synchronization and chaos in an associative neural network with multiple active memories. Chaos: An Interdisciplinary Journal of Nonlinear Science. 2003; 13(3):1090–1104. 10.1063/1.1602211 10.1063/1.1602211 12946202

[60] Terman D, Wang D. Global competition and local cooperation in a network of neural oscillators. Physica D: Nonlinear Phenomena. 1995; 81(1-2):148–176. 10.1016/0167-2789(94)00205-5

[61] Meier M, Haschke R, Ritter HJ. Perceptual grouping through competition in coupled oscillator networks. Neurocomputing. 2014; 141:76–83. 10.1016/j.neucom.2014.02.011

[62] Yu G, Slotine JJ. Visual grouping by neural oscillator networks. IEEE Transactions on Neural Networks. 2009; 20(12):1871–1884. 10.1109/TNN.2009.2031678 19914896 10.1109/TNN.2009.2031678

[63] Horn D, Usher M. Excitatory–inhibitory networks with dynamical thresholds. International Journal of Neural Systems. 1990; 1(03):249–257. 10.1142/S0129065790000151

[64] Horn D, Sagi D, Usher M. Segmentation, binding, and illusory conjunctions. Neural Computation. 1991; 3(4):510–525. 10.1162/neco.1991.3.4.510 10.1162/neco.1991.3.4.510 31167334

[65] Horn D, Opher I. The importance of noise for segmentation and binding in dynamical neural systems. International Journal of Neural Systems. 1996; 7(04):529–535. 10.1142/S0129065796000518 8968844 10.1142/s0129065796000518

[66] Breve FA, Zhao L, Quiles MG, Macau EE. Chaotic phase synchronization and desynchronization in an oscillator network for object selection. Neural Networks. 2009; 22(5-6):728–737. 10.1016/j.neunet.2009.06.027 19595565 10.1016/j.neunet.2009.06.027

[67] Sakai K, Rowe JB, Passingham RE. Active maintenance in prefrontal area 46 creates distractor-resistant memory. Nature Neuroscience. 2002; 5(5):479 10.1038/nn846 11953754 10.1038/nn846

[68] Oberauer K. Declarative and procedural working memory: Common principles, common capacity limits? Psychologica Belgica. 2010; 50(3-4):3–4. 10.5334/pb-50-3-4-277

[69] Garavan H. Serial attention within working memory. Memory & Cognition. 1998; 26(2):263–276. 10.3758/BF03201138 9584434 10.3758/bf03201138

[70] Oberauer K. Selective attention to elements in working memory. Experimental Psychology. 2003; 50(4):257 10.1026//1618-3169.50.4.257 14587173 10.1026//1618-3169.50.4.257

[71] Kessler Y, Meiran N. Two dissociable updating processes in working memory. Journal of Experimental Psychology: Learning, Memory, and Cognition. 2008; 34(6):1339 10.1037/a0013078 18980398 10.1037/a0013078

[72] Pertzov Y, Husain M. The privileged role of location in visual working memory. Attention, Perception, & Psychophysics. 2014; 76(7):1914–1924. 10.3758/s13414-013-0541-y 10.3758/s13414-013-0541-y PMC4212176 24027033

[73] Harrison WJ, Bays PM. Visual working memory is independent of the cortical spacing between memoranda. Journal of Neuroscience. 2018; p. 2645–17. 10.1523/JNEUROSCI.2645-17.2017 PMC5864153 29459370

[74] Doughty J, Garner W. Pitch characteristics of short tones. II. Pitch as a function of tonal duration. Journal of Experimental Psychology. 1948; 38(4):478. 18874604 10.1037/h0057850

[75] Ekman G, Berglund B, Berglund U. Loudness as a function of the duration of auditory stimulation. Scandinavian Journal of Psychology. 1966; 7(1):201–208. 10.1111/j.1467-9450.1966.tb01354.x 5915856 10.1111/j.1467-9450.1966.tb01354.x

[76] Sokolov A, Lutzenberger W, Pavlova M, Preissl H, Braun C, Birbaumer N. Gamma-band MEG activity to coherent motion depends on task-driven attention. Neuroreport. 1999; 10(10):1997–2000. 10.1097/00001756-199907130-00001 10424663 10.1097/00001756-199907130-00001

[77] Börgers C, Kopell NJ. Gamma oscillations and stimulus selection. Neural Computation. 2008; 20(2):383–414. 10.1162/neco.2007.07-06-289 18047409 10.1162/neco.2007.07-06-289

[78] Borisyuk GN, Borisyuk RM, Khibnik AI, Roose D. Dynamics and bifurcations of two coupled neural oscillators with different connection types. Bulletin of Mathematical Biology. 1995; 57(6):809–840. 10.1007/BF02458296 8528157 10.1007/BF02458296

