# Supplementary material for: Oscillations in working memory and neural binding: A mechanism for multiple memories and their interactions
Source: PLoS Comput Biol. 2018 Nov 12;14(11):e1006517. doi: 10.1371/journal.pcbi.1006517 (PMC6258380; doi:10.1371/journal.pcbi.1006517)
Supplement: S1 Text — (PDF) [file pcbi.1006517.s001.pdf]

Supporting Information: S1 Text.
Oscillations in working memory and neural binding: a mechanism for multiple memories and their interactions

Jason E. Pina, Mark Bodner, Bard Ermentrout

Mean field model motivation and spiking network comparison

Consider a population of N *all-to-all* coupled excitatory and inhibitory noisy quadratic integrate-and-fire (QIF) neurons. For simplicity, we use the same number of excitatory and inhibitory neurons. The network has the form:

$$\begin{aligned}\frac{dV_{e,j}}{dt} &= I_e + \sigma_e \xi_{e,j}(t) + V_{e,j}^2 \\ \frac{dV_{i,j}}{dt} &= I_i + \sigma_i \xi_{i,j}(t) + V_{i,j}^2\end{aligned} \quad (1)$$

where ξ are independent white noise processes and the drives include the synaptic interactions and inputs, $I_{e,i}^0(t)$:

$$\begin{aligned}I_e &= I_e^0(t) - \theta_e + a_{ee}\frac{1}{N}\sum_{k=1}^{N} u_k - a_{ei}\frac{1}{N}\sum_{k=1}^{N} v_k + a_{en}h(V_{e,j})\frac{1}{N}\sum_{k=1}^{N} n_k \\ I_i &= I_i^0(t) - \theta_i + a_{ie}\frac{1}{N}\sum_{k=1}^{N} u_k - a_{ii}\frac{1}{N}\sum_{k=1}^{N} v_k + a_{in}h(V_{i,j})\frac{1}{N}\sum_{k=1}^{N} n_k.\end{aligned}$$

For the QIF mode, when $V(t^-) = +\infty$, it is reset to $V(t^+) = -\infty$. (In simulations, we replace $\pm\infty$ by ± 100.) We use current-based interactions rather than conductance-based as they are easier to reduce to a mean-field [1]. The NMDA current

(subscripted n) has an additional voltage dependence [3]:

$$h(V) = 1/(1 + \exp(-0.062(V - 60))[Mg]/3.57)$$

where $[Mg]$ is the extracellular magnesium concentration (set to either 1 or 0 in this paper). (We note that the voltage is shifted by +60, since our simple QIF model has a resting potential at around 0 as opposed to -60.) The synaptic gating variables for AMPA (u_j) and GABA (v_j) obey simple first-order dynamics:

$$\begin{aligned} \tau_e u_j' &= -u_j + \zeta \sum_k \delta(t - t_{jk}^e) \\ \tau_i v_j' &= -v_j + \zeta \sum_k \delta(t - t_{jk}^i) \end{aligned}$$

where ζ is a constant that we will choose to match the simulations and $t_{jk}^{e,i}$ are the kth spikes of the jth neuron. We use a simple first-order voltage-gated model of Golomb et al. [2] for the NMDA synapses:

$$\frac{dn_j}{dt} = -n_j/\tau_n + a_n/(1 + \exp(-(V_{e,j} - V_{th})/V_{shp}))(1 - n_j) \tag{2}$$

with $\tau_n = 144, V_{th} = 60, a_n = 7, V_{shp} = 5$.

Since coupling is all-to-all, we let $\bar{u} = (1/N)\sum_j u_j$, $\bar{v} = (1/N)\sum_j v_j$, and $\bar{n} = (1/N)\sum_j n_j$ Thus, for the AMPA and GABA we get:

$$\begin{aligned} \tau_e \bar{u}' &= -\bar{u} + \zeta(1/N)\sum_{j,k} \delta(t - t_{jk}^e) \\ \tau_i \bar{v}' &= -\bar{v} + \zeta(1/N)\sum_{j,k} \delta(t - t_{jk}^i). \end{aligned}$$

We observe that in the limit as N gets large, the sums approach the mean firing rate of the neurons in each population given the total inputs, I_e, I_i. For the NMDA, we obtain:

$$\frac{d\bar{n}}{dt} = -\bar{n}/\tau_n + \frac{1}{N}\sum_j (a_n/(1 + \exp(-(V_{e,j} - V_{th})/V_{shp}))(1 - n_j))$$

At this point, we make one of our main approximations. Since function $R(V) = 1/(1 + \exp(-(V - V_{th})/V_{shp}))$ is close to 0 when the neuron is not firing, but

grows rapidly when it is firing, we will approximate its average by the average excitatory activity, $\bar{u}$ raised to a power $p > 1$ to account for the threshold-like behavior and, thus, obtain the following:

$$\frac{d\bar{n}}{dt} = -\bar{n}/\tau_n + a_n C_2 \bar{u}^p (1 - \bar{n})$$

with C_2 chosen to match the amplitude of $\bar{n}$ for the spiking model.

To obtain the firing rate, consider the mean first passage time for a noise driven QIF model,

$$dV = (V^2 + I)dt + \sigma dW, \qquad V(0) = -\infty,$$

where σ is the noise and I is the drive. The expected time $T(I, \sigma)$ for $V(t)$ to reach $+\infty$ leads to the expected firing rate, $\nu(I, \sigma) = 1/T$. For the zero noise case, $\nu = \sqrt{[I]_+}/\pi$, where $[I]_+$ is the positive part of I. With noise, this rate can be closely approximated by the nonlinearity:

$$f(I) = \frac{1}{\pi}\sqrt{I/(1 - \exp(-\beta I))},$$

where β is chosen to best fit for a given σ [4].

With this approximation of the noisy firing rate, we get the approximate equations for $\bar{u}, \bar{v}$:

$$\begin{aligned} \tau_e \bar{u}' &= -\bar{u} + \zeta f(I_e) \\ \tau_i \bar{v}' &= -\bar{v} + \zeta f(I_i), \end{aligned}$$

where

$$\begin{aligned} I_e &= I_e^0(t) - \theta_e + a_{ee}\bar{u} - a_{ei}\bar{v} + a_{en}\bar{n}h(V_{e,j}) \\ I_i &= I_i^0(t) - \theta_i + a_{ie}\bar{u} - a_{ii}\bar{v} + a_{in}\bar{n}h(V_{i,j}). \end{aligned}$$

We still have the function $h(V)$ to deal with, and since its main effect is to reduce the effective strength of the NMDA current, we will treat is as a constant, C_1. In particular, with zero $[Mg]$, we have $h(V) = 1$ and $C_1 = 1$. If we choose $\zeta = \pi$, then, the equations for $\bar{u}, \bar{v}$ are exactly the same as those that we have analyzed throughout the paper.

Putting all these parts together, we obtain the mean-field model of the paper:

$$\begin{aligned}
\tau_e \bar{u}' &= -\bar{u} + \zeta f(I_e^0(t) + a_{ee}\bar{u} - a_{ei}\bar{v} + C_1 a_{en}\bar{n} - \theta_e) \\
\tau_i \bar{v}' &= -\bar{v} + \zeta f(I_i^0(t) + a_{ie}\bar{u} - a_{ii}\bar{v} + C_1 a_{in}\bar{n} - \theta_i) \\
\bar{n}' &= -\bar{n} + C_2 a_n \tau_n \bar{u}^p (1 - \bar{n})
\end{aligned}$$

where the two constants, C_1, C_2 come from our approximations of the voltage dependences in the NMDA synapses and currents.

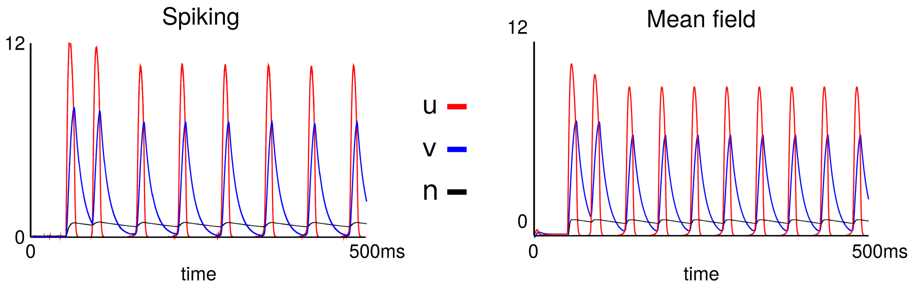

Fig 1. Comparison of the spiking model with the mean field model.
Left: The average of u_j, v_j, n_j for the full spiking model with $[Mg] = 1mM$, $a_{en} = 40$, $a_{in} = 0$, $\zeta = \pi$, $a_n = 7$. Stimulus is a 50ms pulse given at $t = 50$. Right: The mean field model with $C_1 a_{en} = 4$, $C_2 a_n \tau_n = 2$.

Fig 1 shows a simulation of the spiking model with 200 excitatory and 200 inhibitory cells and all parameters as in the paper except as indicated in the text and figure caption in this appendix. The shapes and amplitudes are quite close, although the frequency is faster in the mean field than in the spiking model. We have shown in this appendix that we can find a spiking model that will generate the same dynamics as our Wilson-Cowan type system. Furthermore, we have given a heuristic (via two approximations) way to incorporate NMDA into the mean field model that closely matches the dynamics of the spiking model on which it is based.

References

1. Ermentrout B. Reduction of conductance-based models with slow synapses to neural nets. Neural Computation. 1994; 6(4):679–695.

2. Golomb D, Amitai Y. Propagating neuronal discharges in neocortical slices: Computational and experimental study. Journal of Neurophysiology. 1997; 78(3):1199–1211.

3. Dayan P, Abbott LF. Theoretical Neuroscience. Vol. 806. Cambridge, MA: MIT Press; 2001.

4. Ermentrout GB, Terman DH. Mathematical Foundations of Neuroscience. Vol. 35. Springer Science & Business Media; 2010.
