# Supplementary material for: Oscillations in working memory and neural binding: A mechanism for multiple memories and their interactions
Source: PLoS Comput Biol. 2018 Nov 12;14(11):e1006517. doi: 10.1371/journal.pcbi.1006517 (PMC6258380; doi:10.1371/journal.pcbi.1006517)
Supplement: S2 Text — (PDF) [file pcbi.1006517.s002.pdf]

Supporting Information: S2 Text. Oscillations in working memory and neural binding: a mechanism for multiple memories and their interactions

Jason E. Pina, Mark Bodner, Bard Ermentrout

The u, v, n system

Here we examine the mechanisms by which persistent steady state and oscillatory behaviors arise in our model when the excitatory neurons are excited by a brief input stimulus. The NMDA dynamics ensure NMDA stays bounded within $[0, 1]$ (given initial conditions in the same interval). The null surface for NMDA is sigmoidal ($n = \frac{a_n \cdot u^p}{1+a_n \cdot u^p}$), suggesting the possibility of bistability between a low state and a high state. Indeed, the NMDA allows for latch-like behavior of the system [1], so that low NMDA activity coincides with low u and v values (or low average values in the oscillatory case), and high (average for the oscillatory case) NMDA coincides with higher u and v values. Although Eq (1) in the main text describes a three-dimensional system when $N = 1$, we may gain some intuition for the observed dynamics by fixing n at a constant level (since it evolves slowly and changes little compared to u and v) and examining the reduced $u - v$ system. Thus, changing n in this planar system is equivalent to changing $\theta_{e,i}$ in tandem in the original system. Foregoing the stimulus, the projected system is:

$$\begin{aligned} u' &= -u + f(a_{ee} \cdot u - a_{ei} \cdot v + a_{en} \cdot n - \theta_e) \\ \tau_i \cdot v' &= -v + f(a_{ie} \cdot u - a_{ii} \cdot v + a_{in} \cdot n - \theta_i) \end{aligned} \quad (1)$$

We may now examine the nullclines in Eq (1). As we see in Fig 1, for low n (Fig 1A) the v-nullcline intersects the left branch of the u nullcline, while for high n (Fig 1B), the

v-nullcline intersects the middle branch of the u-nullcline. Thus, the low n case results in a stable fixed point, and the high n case results in a fixed point at somewhat higher u and v that may be stable or unstable, depending on τ_i. For low τ_i (e.g., $\tau_i = 1$), this high fixed point is stable; for higher τ_i, a stable oscillation emerges, as suggested in the example trajectories in Fig 1B. We note that the "high" point in this case still corresponds to low u and v values, though in the oscillatory case this can still lead to large maxima, as we see in Fig 1B, e.g. The nullcline intersection can change based on parameter values, however. Lowering a_{ei} to 8, e.g., results in a middle-branch intersection at much higher u and v values, as suggested by Fig 2A. Therefore, depending on the values of the other parameters, varying n (still as a parameter) can allow such a two-dimensional system to switch between low- and high-activity states that can be either steady-state or oscillatory.

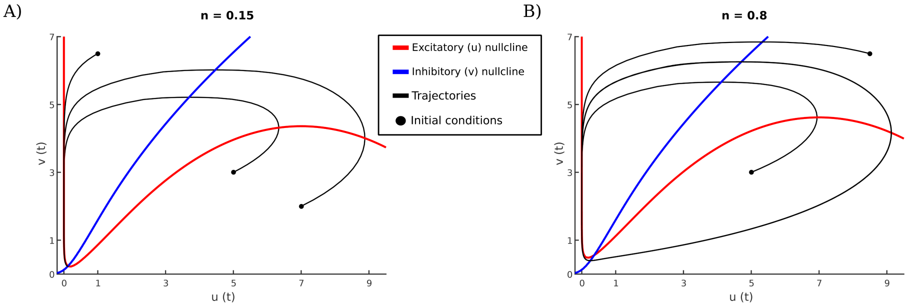

Fig 1. Nullclines and example trajectories for Eq (1).
Parameter values are as given in *Methods* of the main text. (A) For lower NMDA (n) values, the v-nullcline intercepts the left branch of the u-nullcline, resulting in a stable fixed point. (B) For higher NMDA values, the v-nullcline intercepts the middle branch of the u-nullcline; the fixed point is stable for low τ_i values and unstable for larger τ_i values. Here, $\tau_i = 12$, so that the fixed point is unstable and the system has a stable limit cycle, as suggested by the example trajectories.

In the full three-dimensional system, we set up our u and v nullsurfaces so that their low-n and high-n cross-sections intersect as the corresponding 2-dimensional nullclines do. As the NMDA nullsurface is sigmoidal, it can act as a dynamic latch, so that once n is excited enough (via the $s(t) \to u \to n$ path), the system is attracted to the high state. As we just mentioned above, the existence of bistability is, of course, dependent on system parameters; for example, we see that two stable fixed points exist for a range of a_{ei} values in Fig 2A. As τ_i increases, the high fixed point destabilizes and a limit cycle is born via a subcritical Hopf bifurcation (Fig 2B). While this allowed us to easily find

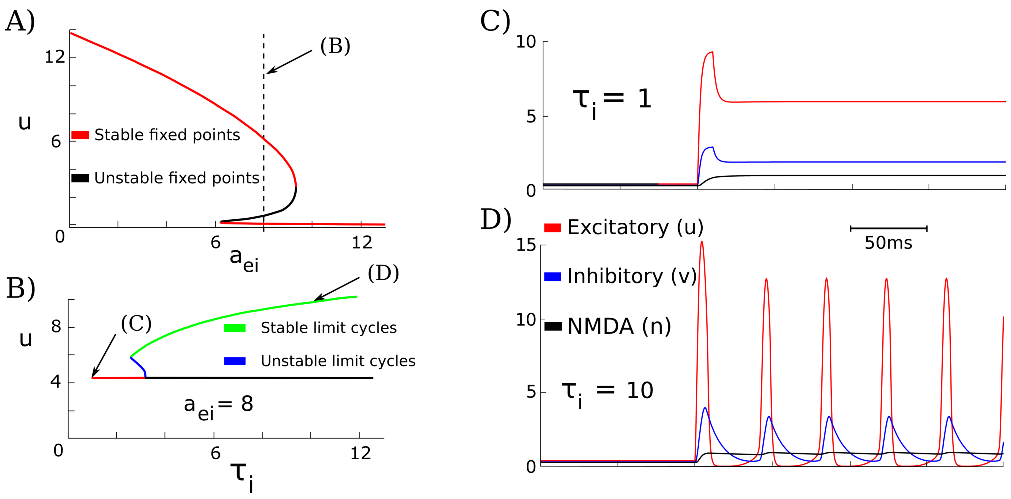

Fig 2. Bifurcations and dynamics for one population for Eq (1) in the main text.
(A) There are open sets of parameters, such as a_{ei}, that allow for bistability between low states and high states. Here, $\tau_i = 1$. (B) Increasing τ_i leads to a subcritical Hopf bifurcation of the high fixed point. (C), (D) display the dynamics for $a_{ei} = 8$ and different τ_i, leading to bistability of fixed points (C) or limit cycles (D).

parameters with which both a stable low fixed point and a stable high, oscillatory, state coexist, we ended up using $a_{ei} = 10$, not 8 as in Fig 2B. For $a_{ei} = 10$, the oscillations are lost as a fold of limit cycles as in Fig 2B as τ_i decreases, but there is no Hopf; rather, we end up with an isola of limit cycles as in Fig 3A in the main text. If τ_i decreases beyond the left fold, only the low steady state stably exists.

We may gain further intuition for how the separation of timescales allows for oscillations. Once the excitatory stimulus kicks the AMPA population sufficiently, it begins an excursion around phase space. In turn, it excites the inhibitory population, which then chases the AMPA population, curtailing its growth. As the inhibitory timescale is slower than that of the excitatory population ($\tau_i > \tau_e = 1$), the inhibition eventually wins out, quenching the activity of the AMPA population, as we see in Fig 2D. This is why the NMDA population is important: The NMDA is excited by the AMPA, but decays much slower than the inhibition ($\tau_n > \tau_i$), allowing the NMDA to outlast the upstroke of the inhibition. By doing so, the NMDA can then re-excite the AMPA population once the inhibition is sufficiently low, producing the observed wavetrain in Fig 2D.

Thus, if τ_n is too small, the NMDA population will increase rapidly with AMPA, but

will then decay too quickly to maintain the activity of the AMPA population after the inhibitory population has quenched it. Clearly the same problem will occur if τ_i is too large. Therefore, the ratio $\frac{\tau_i}{\tau_n}$ must be small enough. However, if τ_i is too small, two possibilities emerge: (1) the inhibition (v) decays too quickly to quench the excitatory population, in which case activation will lead to a stable up state; (2) the inhibition activates very rapidly, quenching the excitatory activity before it increases enough from its baseline levels, in which case no activation occurs: only the low steady state is stable. For our parameter choices, decreasing τ_i leads to (2), as we mentioned above. In either case, the initial phase space excursion that occurs in the oscillatory case cannot take place at all. Instead, after receiving a stimulus the excitation will either settle at the high fixed point in the case of (1) with a strong enough stimulus (as we can see in Fig 2C), or else it will simply decay back to the low steady state. Hence, we now have some intuition to understand the general shapes of the bifurcation curves in Fig 3B, 3C, and 3D in the main text.

References

1. Lisman JE, Fellous JM, Wang XJ. A role for NMDA-receptor channels in working memory. Nature Neuroscience. 1998; 1(4):273–275.
