# Supplementary material for: Oscillations in working memory and neural binding: A mechanism for multiple memories and their interactions
Source: PLoS Comput Biol. 2018 Nov 12;14(11):e1006517. doi: 10.1371/journal.pcbi.1006517 (PMC6258380; doi:10.1371/journal.pcbi.1006517)
Supplement: S3 Text — (PDF) [file pcbi.1006517.s003.pdf]

Supporting Information: S3 Text. Oscillations in working memory and neural binding: a mechanism for multiple memories and their interactions

Jason E. Pina, Mark Bodner, Bard Ermentrout

Weak coupling analysis

Using the parameters shown in *Methods* in the main text for a single local $u-v-n$ circuit, we can perform a weak coupling analysis (WCA) around the limit cycle solution that appears in the up state. That is, since the coefficients that we use tend to be small compared to the self coupling, the WCA can provide some insights into the possible patterns when M groups are oscillating. The results should hold for any size network, since the "down" circuits contribute very little to the interactions in the groups that are oscillating. To perform the WCA, we first see that

$$f(a_{ee}u + a_{en}n - a_{ei}v - \theta_e - \epsilon\hat{v}) \approx f(I_e) - \epsilon\, f'(I_e)\hat{v},$$

where $I_e = a_{ee}u + a_{en}n - a_{ei}v - \theta_e$, and with similar terms for other types of coupling. Recall that in WCA, we reduce

$$X_i' = F(X_i) + \epsilon G_i(X_1, \ldots, X_N), \qquad i = 1, \ldots, N$$

to a set of equations of the form:

$$\theta_i' = 1 + H_i(\theta_1 - \theta_i, \ldots, \theta_N - \theta_i).$$

To achieve this reduction, we assume there is a $T-$periodic limit cycle solution, $U' = F(U)$, and let $Z(t)$ be the unique solution (adjoint solution) to

$Z' = -(D_X F(U))^T$, with $U'(t) \cdot Z(t) = 1$. The phase interaction functions H_i are defined as

$$H_i(\phi_1, \dots, \phi_N) = \frac{1}{T} \int_0^T Z(t) \cdot G_i(U(t + \phi_1), \dots, U(t + \phi_N)).$$

Since all coupling is summed up, we only need to compute the interaction with one other circuit. Thus, we need to compute the adjoint solution and the basic limit cycle. Letting $(u(t), v(t), n(t))$ be the basic limit cycle for the isolated population and $(u^*(t), v^*(t), n^*(t))$ be the corresponding adjoint solution, we compute the following four interaction functions:

$$\begin{aligned}
H_{ee}(\phi) &= \frac{1}{T} \int_0^T u^*(t) f'(I_e(t))[a_{ee} u(t+\phi) + a_{en} n(t+\phi)]\ dt \\
H_{ei}(\phi) &= -\frac{1}{T} \int_0^T u^*(t) f'(I_e(t)) a_{ei} v(t+\phi)\ dt \\
H_{ie}(\phi) &= \frac{1}{T} \int_0^T v^*(t) f'(I_i(t))[a_{ie} u(t+\phi) + a_{in} n(t+\phi)]\ dt \\
H_{ii}(\phi) &= -\frac{1}{T} \int_0^T v^*(t) f'(I_i(t)) a_{ii} v(t+\phi)\ dt.
\end{aligned}$$

Once we have the interaction functions, we can study the dynamics of the oscillators when weakly coupled. We form the composite function:

$$H(\phi) = c_{ee} H_{ee}(\phi) + c_{ei} H_{ei}(\phi) + c_{ie} H_{ie}(\phi) + c_{ii} H_{ii}(\phi).$$

The coupled phase equations satisfy

$$\theta_i' = \sum_{j \neq i} H(\theta_j - \theta_i),$$

where i varies from 1 to N active groups. To determine the locked patterns, we reduce the dimension to $N-1$ by setting $\theta_1 = 0$ and subtracting θ_1' from the remaining $N-1$ equations:

$$\psi_i' = \sum_{j \neq i} H(\psi_j - \psi_i) - \sum_{j > 1} H(\psi_j).$$

Here, ψ_i represents the phase relative to θ_1. Stable fixed points of this $(N-1)-$ dimensional system correspond to the attracting dynamics of the weakly coupled system.

Active	EE	EI	IE	II
2	S, A	S, A	S, A	N
3	S	S, L, C	S, L, C	C
4	S	S, CS, C, L3	S, CS, C, L	C

Table 1. Weak coupling summary of the dynamics for up to 4 active groups. The states are synchronous (S), anti-phase (A), nonsynchronous (N), clustered (C), symmetric cluster (CS), splay (L), and semi-splay (L3). By synchronous, we mean that all the oscillators fire together in-phase; anti-phase means the two oscillators fire a half cycle apart; clustered means that two groups of oscillators form that are synchronous within the group and out-of-phase between groups; symmetric clusters mean that there are equal numbers in each group; nonsynchronous means neither synchronous nor anti-phase; splay is the state: $0,\ 1/N,\ 2/N,\ \ldots,\ (N-1)/N$; semi-splay is a state where 2 oscillators are synchronized and the other two are out of phase but not synchronized themselves. Thus, A, L, and N correspond to the out-of-phase (OP) oscillations described in the main text while C, CS, and L3 correspond to the mixed-phase (MP) oscillations.

Table 1 summarizes the attractors for up to 4 active groups when each of the individual coupling terms are set to 1 and the rest are set to zero. We note that the behavior of EE coupling serves mainly to synchronize and that the EI and IE coupling have similar behavior. Small amounts of EE coupling in addition to EI coupling (what we use in the full model) behave like the EI coupling alone as long as the EE coupling is not too big. For example, with EI coupling and three active groups, WCA predicts that there will be synchrony (S), a splay state (L), and a clustered state (C) with two oscillators synchronized and the third out of phase (see Fig 7 in the main text, where L is indicated as OP and C as MP). Since the coupling is all-to-all and symmetric, all possible permutations of the attractors occur.

This analysis explains many of the interactions we see with active populations. We remark that the analysis is only valid when the coupling is weak enough, but it still manages to include many of the attractors that are seen when there are two or more active populations. We finally note that for 5 or more active populations we only see various clustered states and synchrony with weak coupling; we do not see any splay states. Thus, at least when the coupling is weak, there is limited capacity in the network.
