# Supplementary material for: Oscillations in working memory and neural binding: A mechanism for multiple memories and their interactions
Source: PLoS Comput Biol. 2018 Nov 12;14(11):e1006517. doi: 10.1371/journal.pcbi.1006517 (PMC6258380; doi:10.1371/journal.pcbi.1006517)
Supplement: S4 Text — (PDF) [file pcbi.1006517.s004.pdf]

Supporting Information: S4 Text.
Oscillations in working memory and neural binding: a mechanism for multiple memories and their interactions

Jason E. Pina, Mark Bodner, Bard Ermentrout

Change in OP and S dynamics with varying coupling strengths and synaptic timescales

OP solutions (N=2)

The OP state arises through competition that is facilitated by the mutual inhibition, c_{ei}. For two populations P1 and P2, as we have here, each excitatory component, u_1 and u_2, peaks twice during each oscillation; once at a large value (the large peak) and once at a small value (the small peak) (see Fig 1). Henceforth we will refer to the population that peaks at a large value as the primary population, and the one that peaks at a small value as the secondary population. For example, suppose P1 is the primary population and P2 is the secondary population as in the first half of the oscillation in, e.g., Fig 1B. The downstrokes for both u_1 from its large peak and u_2 from its small peak are caused by the upstroke of v_1. Thus, u_2 (and therefore P2, since our readout is the excitatory component) is kept inactive by the competitive activity from v_1.

What happens as the coupling strengths c_e and c_{ei} change? Several characteristics may become altered, including the maxima and minima that the various components achieve, the period of the oscillation, and the relative phase timings of the components. However, since the populations are coupled together only through the excitatory

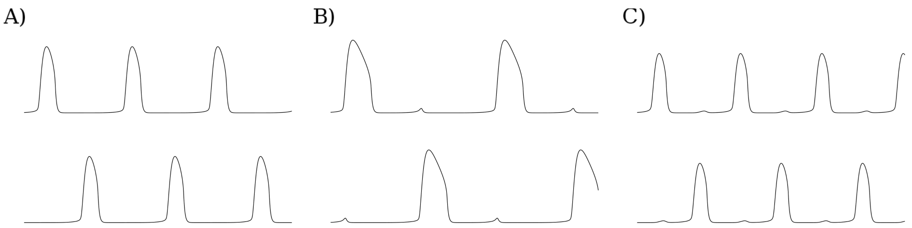

Fig 1. Example traces of OP states for $N = 2$ and varying c_e and c_{ei}.
As c_e and c_{ei} increase, the small peak becomes more pronounced. (A) $c_e = 0,\ c_{ei} = 0.01$. Here no small peak occurs. (B) $c_e = 0,\ c_{ei} = 0.54$. The primary population (top plot at the beginning of the timecourse) begins to rise again after its first large excursion from its baseline value, but the secondary (bottom plot at the start of the trace) population comes on and is seen to stop the first population in its tracks, so that only a small, secondary excursion occurs. (C) $c_e = 0.055,\ c_{ei} = 0.0855$. The presence of the excitatory coupling from nonzero c_e pushes the small peak in such a way as to be more coincident with the large peak of the alternate population; however, the inhibition due to c_{ei} still suppresses it, again only allowing for a small excursion from baseline.

components, perhaps the most important change is the increase in the amplitude of the small peak. Once the the small peak increases too much, the secondary population may no longer be suppressed, becoming active and perhaps synchronizing with the primary population. This is manifested as a bifurcation (see Fig 6C in the main text). Indeed, in all of the cases we examined, if either c_e or c_{ei} increases beyond the bifurcation values, the network transitions to synchrony in numerical simulations. We first look at varying c_e and then the more complicated and somewhat paradoxical picture of varying c_{ei}.

Fixed c_{ei}, varying c_e

Fixing c_{ei} and varying c_e results in (1) slightly decreased period; (2) decreased large peak; (3) increased small peak (Table 1). Suppose again that P1 is the primary population and P2 is the secondary population, so that $u_1 > u_2$ in the interval of interest. We first observe that as c_e increases, u_2 receives significantly more excitation, while u_1 receives *less* excitation. This is a direct result of the normalization we have used. In particular, from Eq (2) in the main text we see that for two populations,

$$\widetilde{u}_1 = \frac{u_1 + c_e\, u_2}{1 + c_e}.$$

c_e	Period	Large u peak	Small u peak	v_{max}	v_{min}	n_{min}
0.00	92	10.0	0.05	7.5	0.15	0.59
0.05	89	9.3	0.64	6.8	0.17	0.60

Table 1. OP state changes with c_e.
An example of the changes to the OP state for $N = 2$ that occur as c_e increases while c_{ei} remains fixed at 0.3.

c_{ei}	Period	Large u peak	Small u peak	v_{max}	v_{min}	n_{min}
0.00	49	9.2	N/A	6.3	0.39	0.77
0.54	114	10.4	0.64	8.2	0.14	0.53

Table 2. OP state changes with c_{ei}.
An example of the main changes to the OP state for $N = 2$ that occur as c_{ei} increases while c_e remains fixed 0. Note that the value for the small u peak indicates "not applicable" since the peak in the secondary excitatory component only occurs for large enough c_{ei} and c_e.

Differentiating $\widetilde{u}_1$ with respect to c_e shows that it monotonically decreases as c_e increases if $u_1 > u_2$, as we have assumed. As a result, u_1's maximum decreases, so that the width of the pulse of u_1 decreases, whereas the length of its quiescent phase experiences almost no change. Thus, the small decrease in period is mostly due to the decrease in the amplitude of the large peak. For nonzero c_{ei}, the increase in the maximum of u_2 in turn further excites v_2, so that u_1 experiences greater inhibition. Thus, in addition to the effect just now described, increasing c_e results in a lower maximum for u_1 since v_2 inhibits u_1 more when c_{ei} is nonzero. In either case, once the maximum of the secondary u becomes too large relative to that of the primary u, the splay state is lost to synchrony.

Fixed c_e, varying c_{ei}

As c_{ei} increases, we observe the following changes: (1) the peak of the large amplitude changes, increasing monotonically until just before bifurcation; (2) the small peak changes, generally increasing monotonically; (3) the period of the oscillation changes, increasing monotonically until just before bifurcation; (4) the inhibition generally peaks at larger values and decays to smaller values; (5) the NMDA generally decays to lower levels (Table 2). There are some subtle differences when c_e is low or high, and we discuss each in turn.

When c_e is low or zero, the effects of increasing c_{ei} are straightforward. We again suppose P1 is the primary population and P2 is the secondary population. For simplicity we will focus on $c_e = 0$. The excitatory component of the primary population, u_1, cannot begin its larger upstroke until $\widetilde{v}_1$ is sufficiently low. Since v_1 is very low before u_1 begins its large upstroke, v_2 provides most of the inhibition that keeps u_1 low before its large upstroke. However, we again look at the coupling term:

$$\widetilde{v}_1 = \frac{v_1 + c_{ei}\, v_2}{1 + c_{ei}}, \tag{1}$$

Differentiating with respect to c_{ei}, we see that $\widetilde{v}_1$ increases (for fixed v_1 and v_2) with increasing c_{ei} when $v_2 > v_1$. Thus, as c_{ei} increases, both v_1 and v_2 must decay to lower values before releasing u_1.

For identical reasons, u_2 receives less inhibition at the beginning of the large upstroke of u_1 as c_{ei} increases. That is, $\widetilde{v}_2$ decreases (for fixed v_1 and v_2) as c_{ei} increases when $v_2 > v_1$. This leads to lower inhibition for u_2 as it begins its small upstroke, allowing it to peak at a higher value (we note that n_2 has also decayed to a lower value, leading to less excitation for u_2; however, since a_{en} is much smaller than a_{ei}, this effect is much smaller).

In summary, larger c_{ei} requires lower inhibitory component values in order for the excitatory components to activate, leading to larger amplitudes of the small peak, a result that eventually destabilizes the antiphase solution. In this scenario, it seems that the increases in the period and the amplitude of the large peak are secondary effects.

We note that there is some competition between the effects of c_{ei}. On the one hand, larger c_{ei} may allow a smaller amount of inhibition of one population to quench the activity of the other; on the other hand, for reasons outlined in the above paragraph, larger c_{ei} can increase the amplitudes of the smaller peaks. Thus, depending on, for example, the precise phase timings of the various components of the two populations, larger c_{ei} could either quench or enhance the activity of the secondary population.

When c_e is larger, we observe exactly these competitive effects as c_{ei} increases. In particular, while the large peak increases monotonically until just before bifurcation, the small peak first increases, then decreases, then increases again. This pattern leads

directly to the nonmonotonic behavior of curve (iv) in Fig 6C in the main text. That is, for some c_e values, the OP state is lost, regained, and lost again as folds of limit cycles with increasing c_{ei}. This appears to be due directly to the nonmonotonic behavior of the small peak for the reasons we described above. In particular, the c_{ei} values at which the amplitude of the small peak begins to decrease converge to the minimum of curve (iv) in Fig 6C in the main text. We note that this competition that leads to the nonmonotonic curve depends on the particular parameters. We have explored other parameter sets, e.g., with which this curve simply decreases monotonically as c_{ei} increases.

Synchronous solutions, varying c_{ei}

In Fig 4D in the main text, we see a general tendency for increasing c_{ei} to increase the interval of τ_i for which we obtain stable M-S solutions, where $M \in \{1, ..., N\}$. Here we provide some heuristic reasoning for why this may be the case.

We first note that when $M = N$, as we described at the beginning of *Maximum S populations* of the main text, the network oscillates as if there were only one population, although the c_{ei} value somewhat changes the range of parameters that allows for this oscillation to exist stably. This is expected, as any perturbation of one of the populations will mean it will feel inhibition from the remaining $M - 1$ populations, and will provide inhibition to them as well. In particular though, we note that as c_{ei} changes for this case, the behavior of the solutions do not change at all; neither the period nor any of the amplitudes change with varying c_{ei}. This is not the case for $M \neq N$.

For $M < N$, we begin to observe changes in the period, the amplitudes of the various components, and the waveform of the solutions. Generally speaking, increasing c_{ei} both increases the largest and decreases the smallest τ_i values that admit stable oscillations, as we see in Fig 4D in the main text. While this is not strictly the case for $M = 4$ as the smaller τ_i limit increases very slightly, it indicates the trend. We note that while the cases of $M = 2, ..., N - 1$ may be argued similarly to the case of $M = 1$, there are certain subtleties that somewhat complicate the picture. Since, as we see in Fig 4D in the main text, the range of τ_i increases for the 1-S oscillation in the same way as for $M = 2$ or 3 and nearly in the same way as for $M = 4$, we will focus on the

simplest case of $M = 1$.

As τ_i increases, the period lengthens and the maxima of the excitatory and inhibitory solutions increase. As we explain in S2 Text, if τ_i is too large relative to τ_n, the NMDA will not outlast the inhibition and the oscillations will cease. If τ_i is too small (and we are not in a parameter regime that allows for a stable high steady state; see S2 Text) the inhibition activates very rapidly, quenching the excitatory activity before it increases enough from its baseline levels, in which case no activation occurs: only the low steady state is stable. The periods and amplitudes of the populations also change with c_{ei} as shown in Fig 2. The amplitudes increase monotonically with c_{ei} (although perhaps by very little, as we see in Fig 2 for $\tau_i = 39.8$), while the periods may increase or decrease.

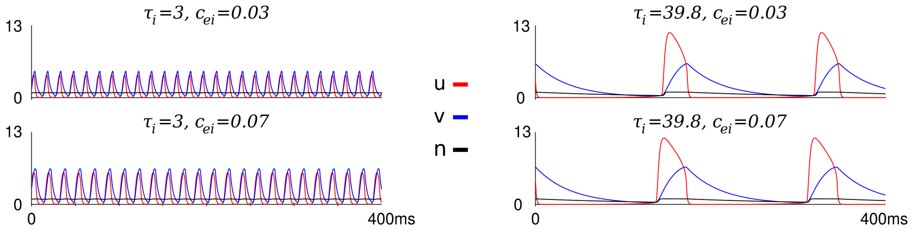

Fig 2. Example traces of one population oscillating with $N = 5$.
In each row, c_{ei} is fixed (and increases as we go from the top row to the bottom row), while τ_i increases from left to right. As τ_i increases with c_{ei} fixed, the period and amplitude increase monotonically and substantially, while as c_{ei} increases with τ_i fixed, the amplitudes increase monotonically, but by much smaller amounts for the given range of c_{ei} values, while the periods show more complicated behaviors. For $\tau_i = 3$ (left 2 plots), the period increases monotonically with c_{ei}, while for $\tau_i = 39.8$ (right 2 plots), the period in fact decreases and then increases as c_{ei} increases from 0.03 to 0.07. For both τ_i values, decreasing c_{ei} just a little bit below 0.03 results in the loss of the oscillations to the low steady state.

The stronger trend with increasing c_{ei} that we observe in Fig 4D in the main text (where c_{ei} increases from 0.03 to 0.07) is the increase in the upper τ_i limit. To see why this might occur, consider $M = 1$, suppose that P1 is the active population, and note that the populations that are inactive are not only at similarly low levels, but in fact are themselves oscillating synchronously at low values. Therefore, the inhibition that u_1 receives is given by Eq (2) in the main text, which we specify for the case of $\widetilde{v}_1$ here:

$$\widetilde{v}_1 = \left(v_1 + c_{ei} \sum_{k=2}^{N} v_k\right) \Big(1 + c_{ei}\ (N-1)\Big)^{-1},$$

which simplifies to

$$\widetilde{v}_1 = \frac{v_1 + c_{ei} v_j}{1 + c_{ei}},$$

where j can be anything in $\{2, ..., N\}$ since, as we mentioned, $\{\mathrm{P}2, ..., \mathrm{P}N\}$ are synchronous. This, of course, is identical to Eq (1), and so the same analysis can be applied as was done in that case. In particular, we note that since P1 is the only active population, u_1 is always (or nearly so) larger than u_j, for $j \in \{2, ..., N\}$. Thus, following the above analysis (where P1 is essentially always the primary population), $\widetilde{v}_1$ *decreases* with increasing c_{ei}, so that u_1 receives *less* inhibition. This exactly explains the increase in the maxima of u_1 with increasing c_{ei} (and, since v_j is excited by u_j, the increase in the maximum of v_1 as well). More relevantly, u_1 needs less excitation from n_1 to maintain the large oscillations for larger c_{ei} values, allowing the oscillations to remain stable for larger τ_i values, as we see in Fig 4D in the main text.

The same explanation holds for low τ_i values. As c_{ei} increases, u_1 receives less inhibition, so that u_1 can maintain its activity for smaller τ_i. For example, suppose $c_{ei} = 0.03$ and $\tau_i = 2.9$, the lower point for $M = 1$ in Fig 4D in the main text. As c_{ei} increases, u_1 receives less inhibition, so that v_1 needs to activate even faster to prevent u_1 from its large excursion from baseline. Thus, τ_i must be lowered still further for $c_{ei} = 0.07$ before P1 will be unable to remain active (down to $\tau_i = 2$ in this example).
